# Synthetic Cationic Peptide IDR-1018 Modulates Human Macrophage Differentiation

**DOI:** 10.1371/journal.pone.0052449

**Published:** 2013-01-07

**Authors:** Olga M. Pena, Nicole Afacan, Jelena Pistolic, Carol Chen, Laurence Madera, Reza Falsafi, Christopher D. Fjell, Robert E. W. Hancock

**Affiliations:** Department of Microbiology and Immunology, University of British Columbia, Vancouver, Canada; University of Central Florida College of Medicine, United States of America

## Abstract

Macrophages play a critical role in the innate immune response. To respond in a rapid and efficient manner to challenges in the micro-environment, macrophages are able to differentiate towards classically (M1) or alternatively (M2) activated phenotypes. Synthetic, innate defense regulators (IDR) peptides, designed based on natural host defence peptides, have enhanced immunomodulatory activities and reduced toxicity leading to protection in infection and inflammation models that is dependent on innate immune cells like monocytes/macrophages. Here we tested the effect of IDR-1018 on macrophage differentiation, a process essential to macrophage function and the immune response. Using transcriptional, protein and systems biology analysis, we observed that differentiation in the presence of IDR-1018 induced a unique signature of immune responses including the production of specific pro and anti-inflammatory mediators, expression of wound healing associated genes, and increased phagocytosis of apoptotic cells. Transcription factor IRF4 appeared to play an important role in promoting this IDR-1018-induced phenotype. The data suggests that IDR-1018 drives macrophage differentiation towards an intermediate M1–M2 state, enhancing anti-inflammatory functions while maintaining certain pro-inflammatory activities important to the resolution of infection. Synthetic peptides like IDR-1018, which act by modulating the immune system, could represent a powerful new class of therapeutics capable of treating the rising number of multidrug resistant infections as well as disorders associated with dysregulated immune responses.

## Introduction

The effectiveness of the innate immune response in eradicating pathogens is determined by numerous processes that work simultaneously or consecutively. One important process in the innate immune response is the local production, or secretion from phagocytes, of host defence peptides (HDP) [1]. In addition to their modest antimicrobial activity, HDPs modulate the immune response to promote the clearance of pathogens, while preventing the deleterious effects of excessive inflammation. In addition, HDPs regulate the transition to adaptive immunity and promote wound healing [1]. Recent research has led to the generation of synthetic peptides that demonstrate enhanced key protective functions such as chemotaxis, wound healing, and immune cell survival, while suppressing pro-inflammatory responses to non-pathological levels [2]. These peptides, termed innate defence regulators (IDRs), enhance the efficiency of the immune response, making them an enticing new anti-infective strategy. They protect in mouse models against many different infections and inflammation and their activity in these models is compromised by treatment with liposomal clodronate indicating that protective activity is dependent on monocytes/macrophages [41], [42]. Of the peptides designed to date, IDR-1018 is a promising candidate based on its minimal cytotoxic activity, ability to significantly reduce LPS-induced cytokine production, ability to promote chemokine production [3], [4] and enhanced resolution of infection and inflammation in animal models [5].

Macrophages are vital components of the innate immune response during health and disease. They respond in a rapid and efficient manner to physiological changes and microbial challenges in the microenvironment, promoting the return to an appropriate homeostatic balance [6], [7]. To accomplish this, macrophages can differentiate, where appropriate towards classically- (M1) or alternatively- (M2) activated phenotypes. M1 macrophages can be induced by Th1 cytokines such as interferon-γ (IFNγ) and TLR ligands like LPS. They are considered to be potent effector cells in inflammatory responses, able to effectively kill microorganisms and tumor cells and produce copious amounts of pro-inflammatory cytokines and specific chemokines. In contrast, M2 macrophages can be induced by Th2 cytokines such as IL4, IL13, IL10, other immune factors such as M-CSF, and consecutive, tolerizing exposures to LPS. They are considered to be primarily involved in tuning inflammatory responses, scavenging debris and apoptotic cells and promoting angiogenesis, tissue remodeling and repair [8], [9], [10], [11]. Although this simple classification of macrophages is practical, it does not account for the vast number of inducing factors and the complexity of the different phenotypes produced by them, leading to many variants of these two basic classes of macrophages. For example, various M2 subsets with different properties have been characterized [12], [13] including M2a macrophages activated by IL4 or IL13, M2b macrophages activated by immune complexes, and M2c macrophages polarized with glucocorticoids or IL10 [13]. Therefore, sometimes choosing a stimulant to study M2 macrophage responses can be difficult. For example, Martinez et al found that stimulation of macrophages with M-CSF, a homeostatic growth factor, lead to the expression of an M2-like transcriptome very similar to that promoted by IL4, suggesting than under basal conditions macrophages default towards an M2 phenotype. Thus M-CSF has been one of the main stimulants chosen to study general M2 responses in many recent studies [14], [15], [16], [17].

Plasticity, a hallmark feature of macrophages, allows them to differentiate into and switch between different phenotypes. However, in certain circumstances, this function is altered and macrophages can be locked into a specific phenotype, leading to pathological conditions such as chronic inflammatory diseases associated with an M1 phenotype, or immunosuppresive disorders associated with an M2 phenotype [18], [19].

The molecular mechanisms that underlie the development of M1 and M2 macrophages, involve a network of molecules that activate specific transcription factors as well as inducing epigenetic and posttranscriptional changes. For instance, NFκ-B and STAT1 are critical transcription factors involved in the induction of M1 macrophages by LPS and IFNγ respectively. NFκ-B and STAT1 subsequently induce the expression of signature M1 pro-inflammatory molecules including TNFα, COX-2, IL12, CCL3 [20], [21]. On the other hand, M2a macrophages induced by IL-4 and IL-13 demonstrate activated STAT6, while M2c macrophages induced by IL10 have activated STAT3. These transcription factors interact and cooperate with other transcription factors such as PPARγ to inhibit M1 associated genes and up-regulate key M2 associated genes such as those encoding mannose receptor, IL10 and TGFβ [22]. Epigenetic regulation is also critical for macrophage differentiation. For example, JMJD3, a H3K27-specific demethylase, is responsible for the differentiation of M2 macrophages in response to M-CSF exposures *in vitro* and host responses to helminth infection *in vivo*. The induced epigenetic changes lead to the activation of essential transcription factors such as IRF4, promoting the up-regulation of M2 signature genes [16], [23].

Based on the role of macrophages/monocytes in IDR peptide mediated protection against infection and initial studies performed on IDR-1018, we hypothesized that IDR-1018 promoted the differentiation of human macrophages towards an immunomodulatory phenotype similar to that of the M2 macrophages. By comparing macrophages differentiated in the presence of IDR-1018 alone, or in combination with an M2-inducing factor M-CSF (referred to here as IDR1018+M2), with those differentiated in the presence of IFNγ or M-CSF, inducing an M1 or M2 phenotype, respectively, we were able to demonstrate that IDR-1018 stimulated a phenotype intermediate between these two extremes.

## Materials and Methods

### Ethics statement, Cells and Reagents

Venous blood was collected from healthy volunteers into heparin-containing Vacutainer tubes (BD Biosciences, San Jose CA) with previous written informed consent obtained from all the volunteers. This procedure and all research done using these samples was carried out in accordance with the guidelines of the UBC Clinical Research Ethics Board (UBC-CREB) and approved under the UBC-CREB# H04-70232.

Peripheral blood mononuclear cells (PBMC) were isolated as described previously [24], [25]. Jurkat cells were obtained from the ATCC (Lymphocytes Human Leukemia J45.01 - CRL-1990) and cultured as described by the ATCC. PBMC were cultured in complete media consisting of RPMI 1640 medium supplemented with 10% (v/v) heat-inactivated Fetal Bovine Serum (FBS), 2 mM L-glutamine, and 25 mM HEPES (all from Invitrogen, Carlsbad CA). All cells were cultivated in a humidified 37°C incubator containing 5% CO_2_.

Lipopolysaccharide (LPS) was obtained from *Pseudomonas aeruginosa* PAO1, strain H103, grown overnight in Luria-Bertani broth at 37° and isolated using the Darveau-Hancock method which gives a highly purified LPS, free of proteins and lipids [26]. Purified LPS samples were quantified using the 2-keto-3-deoxyoctuloosonic acid assay and resuspended in endotoxin-free water (Sigma-Aldrich, Saint Louis MO). LPS was used at a concentration of 10 ng/ml.

IDR-1018 (VRLIVAVRIWRR-CONH2) was synthesized by CPC Scientific (Sunnvale, CA) using solid phase Fmoc chemistry and purified (>95% purity) using reversed phase HPLC. The correct peptide mass was confirmed by mass spectrometry.

### Human macrophage differentiation

Human macrophage differentiation was performed as described previously [10], [17], with some modifications. Briefly, after peripheral blood mononuclear cell isolation in PBS, cells were resuspended in serum-free RPMI medium and plated at 5×10^6^ cells/well in 6 well plates for 30 minutes. Subsequently, media was changed and fresh complete media was added. Twenty four hours later, adherent monocytes were gently washed and treated with the different stimuli as follows: IFNγ at 20 ng/ml (Immunotools, Friesoythe, Germany) for M1 differentiation, M-CSF (Research Diagnostic Inc, Concord, MA) at 10 ng/ml for M2 differentiation and IDR-1018 at 5 ug/ml. Cells were cultured for seven days, with gentle washes and media changes on the second and sixth day, during which treatments were re-added. Finally, on day seven, cells were gently washed and left untreated or challenged with LPS at 10 ng/ml.

### RNA Isolation

RNA was isolated from cell lysates 4 hours post-treatment using the Qiagen RNeasy Isolation Kit (Qiagen, Valencia, CA), as per the manufacturer's instructions, treated with RNase free DNase (Qiagen, Valencia, CA), and eluted in RNase-free water (Ambion, Austin, TX). The RNA concentration was assessed using a NanoDrop spectrophotometer, while RNA integrity and purity was determined by Agilent 2100 Bioanalyzer using RNA Nano kits (Agilent technologies).

### Quantitative real-time PCR (qRT-PCR)

Gene expression was analyzed via qRT-PCR. It was performed using the SuperScript III Platinum Two-Step qRT-PCR kit with SYBR Green (Invitrogen, Carlsbad, CA) as per the manufacturer's instructions, and the ABI Prism 7000 sequence detection system (Applied Biosystems, Carlsbad, California). Briefly, 500 µg of total RNA was reverse transcribed using qScriptTM cDNA Synthesis Kit (Quanta Biosciences, Gaithersburg, MD). PCR was conducted in a 12.5 µl reaction volume containing 2.5 µl of 1/5 diluted cDNA template. A melting curve was performed to ensure that any product detected was specific to the desired amplicon. Fold changes were calculated after normalizing the change in expression of the gene of interest to the housekeeping gene encoding beta-2-microglobulin (B2M), using the comparative Ct method [27] The primers sequences (all from Invitrogen) used for qRT-PCR are presented in Table S1.

### RNA-seq and Analysis

RNA-seq was performed by high-throughput next generation sequencing using the Illumina Genome Analyzer IIx platform. PBMC were initially obtained from 4 healthy donors, followed by monocyte isolation using the EasySep Monocyte Enrichment Without CD16 Depletion Kit. (Stem Cell Technologies, Vancouver, BC) as per manufacturer's instructions. Monocytes were stimulated for 4 hours with 20 µg/ml IDR-1018 and compared to unstimulated monocytes. RNA was then extracted and its quality assessed as described above. For library preparation, 500 ng of total RNA was processed according to the Illumina TruSeq RNA sample preparation guide (Illumina catalogue number FC-122-1002). Briefly, mRNA was purified using poly-dT beads, followed by synthesis of the first and second cDNA strands, end repair addition of a single-A overhang, and ligation of adapters and unique barcodes, as per the manufacturer's instructions. DNA enrichment was carried out via a 15-cycle PCR. Following quantification, 8 pM of dsDNA was used for cluster generation on a CBOT instrument (Illumina, San Diego, CA). RNA sequencing was done on a GAIIx instrument (Illumina), performed as a single read run with 51 amplification cycles. Data processing was carried out in house, using CASAVA to convert raw data and demultiplex to FASTQ sequence files Reads were aligned to the reference genome using Bowtie and Tophat, and then mapped to genes using the Bioconductor package GenomeRanges. Differential gene expression was determined using the edgeR Bioconductor package, and p-values were adjusted for multiple correction using Benjamini-Hochberg (false discovery rate) method. Differentially expressed genes are presented in table S2 and complete RNA sequencing data has been deposited in the Gene Expression Omnibus public database (GSE40131). Transcriptional and bioinformatic analysis of the RNA-seq data was done using system biology tools developed in our laboratory including the InnateDB database (http://www.innatedb.ca) [28], and MetaGEX (http://marray.cmdr.ubc.ca/metagex/). Genes with fold changes of greater than 1.5 and p values <0.05 were considered differentially expressed.

### Enzyme-linked immunosorbent assay (ELISA)

ELISA was performed on supernatants collected at 4 and 24 h post-treatment. These included TNF-α, IL-10 (eBioscience), IP-10, CCL22 (R & D systems), and CCL-3 (Biosource). ELISA assays were performed according to the kit manufacturers' instruction.

### Phagocytosis of apoptotic cells

Phagocytosis of apopototic cells was investigated as described previously [29] with slight changes. Briefly, Jurkat cells were labeled with 0.25 uM CFDA-SE (Invitrogen). Apoptosis was induced by 10 minutes of UV exposure followed by 5 hours of incubation. Apoptotic Jurkat cells were added to differentiated macrophages at a ratio of 10∶1. Macrophages were then gently washed once and detached using trypsin-EDTA. Analysis of phagocytosis was performed using a FACSCalibur system and FlowJo Software, with a CD14^+^ gate used to select for macrophages.

### Statistical Analysis

All treatments were compared to those for M1 macrophage responses, which were used as a control. Statistical significance was determined using a two-tailed Student t-test for paired comparisons using the Prism 4.0 software (*, P<0.05; **, P<0.01; ***, P<0.001).

## Results

### Macrophages differentiated in the presence of IDR-1018 showed an intermediate cytokine response profile when compared to M1 and M2 macrophages

**Figure 1:**
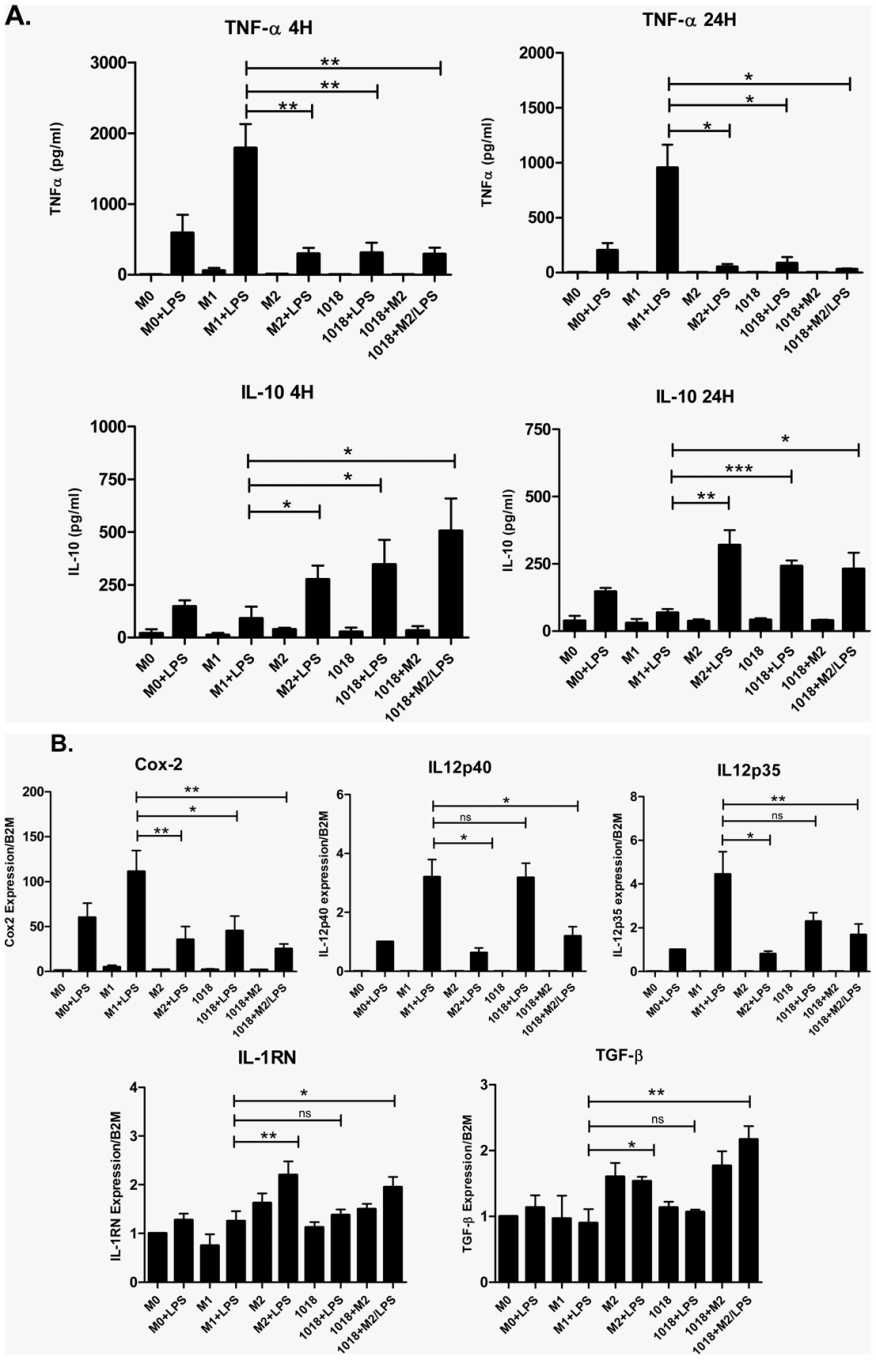
Macrophages differentiated in the presence of IDR-1018 showed an intermediate cytokine response profile when compared to M1 and M2 macrophages. Adherent monocytic cells were differentiated into macrophages in the presence of IFNγ (M1), M-CSF (M2), IDR-1018 alone or in combination with M-CSF (1018+M2), or left untreated (M0). Macrophages were then left unstimulated or stimulated with LPS for 4 or 24 hours after which, the cytokine responses were measured by ELISA (A) and RT-qPCR (B). The data was analyzed for significant differences between the treatments and the M1 phenotype. Mean ± SD results are presented and are representative of 4 biological replicates. ***, P<.0.0001; **, P<0.01; *, P<0.05.

The reduction of pro-inflammatory cytokines and the enhancement of anti-inflammatory mediators is a hallmark of alternatively activated M2 macrophages [30]; therefore we sought to examine this feature in macrophages differentiated in the presence of IDR-1018. To confirm the immunomodulatory effects of IDR-1018, several scrambled synthetic peptides were tested in PBMC to observe chemokine expression (e.g. Figure S1). Conversely natural host defense peptide LL-37 was also employed to observe its effects on macrophage differentiation (Figure S2). In both cases, IDR-1018 showed distinctive responses. Additionally, to select an appropriate inducer of M2 differentiation, we carried out initial experiments using different differentiation protocols, including the one described by Martinez et al [11] using MCSF during the whole process of differentiation and then adding IL-4 as M2 inducer (S Figure S2). However, we elected to utilize a simpler protocol using only M-CSF, which is a known inducer of the M2 phenotype and gave quite similar results (Figure 1). Macrophages differentiated without specific stimulation (M0 cells) and those differentiated in the presence of IFNγ (M1 cells) and M-CSF (M2 cells), were used as controls for comparison with IDR-1018 and IDR1018+M2 (i.e. with added M-CSF) differentiated macrophages. All differentiated macrophages were left untreated or challenged with LPS and cytokine expression was analyzed by ELISA.

IDR-1018 differentiated macrophages responded to LPS stimulation in a complex manner. Some responses were analogous to those of M2 macrophages, but distinct from that of M1 macrophages, in that the pro-inflammatory cytokine TNFα was strongly reduced while the anti-inflammatory cytokine IL10 was highly expressed at both 4 and 24 hours after stimulation (Figure 1A). Likewise, the transcription of another pro-inflammatory mediator, Cox-2, was diminished, as analyzed by RT-qPCR (Figure 1B). Conversely for other inflammatory mediators such as IL-12 subunits, as well as IL-1RN and TGF-β, the responses of IDR-1018 differentiated macrophages were not significantly different when compared to M1 macrophage responses. In contrast, IDR-1018+M2 differentiated macrophages demonstrated similar or stronger responses to those observed for M2 macrophages in reducing all pro-inflammatory mediators while enhancing the anti-inflammatory ones (Figure 1). Overall it appeared that differentiation in the presence of IDR-1018 led to an intermediate phenotype that resembled specific aspects of M1 or M2 macrophages.

### Macrophages differentiated in the presence of IDR-1018 exhibited a chemokine profile different from that of M2 macrophages

**Figure 2:**
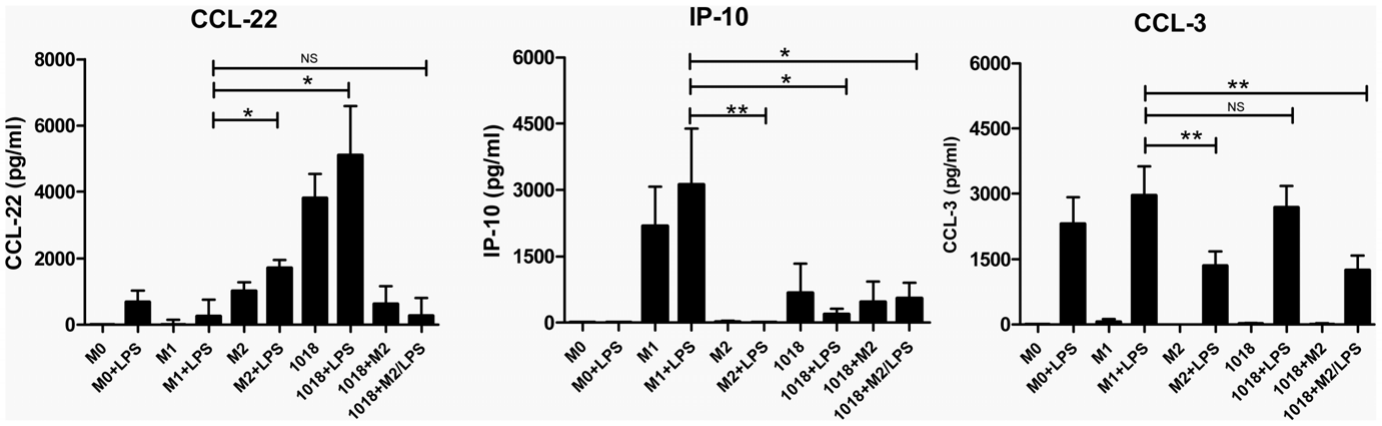
Macrophages differentiated in the presence of IDR-1018 exhibited a chemokine profile different to that of M2 macrophages. Adherent monocytic cells were differentiated into macrophages in the presence of IFNγ (M1), M-CSF (M2), IDR-1018 alone or in combination with M-CSF (1018+M2), or left untreated (M0). These macrophages were then stimulated ± LPS. Twenty four hours post stimulation; the chemokine responses were measured by ELISA and analyzed for significant differences when compared to the M1 phenotype. Raw values were normalized to M0 Macrophages. Mean ± SD results are presented and are representative of 4 biological replicates. **, P<0.01; *, P<0.05.

M1 and M2 macrophages have unique and very characteristic chemokine profiles [31]. For example, M2 macrophages exhibit reduced expression of chemokines such as CCL-3 and IP-10, and higher expression of CCL-22 compared to M1 macrophages. Therefore, the chemokine profile of IDR-1018 differentiated macrophages was examined for these chemokines. IDR-1018 differentiated macrophages presented a profile different from that of M2 macrophages. Although a similar reduction in IP-10 was observed compared to that of M1 macrophages, there was no reduction in CCL3 and a higher production of CCL22 (Figure 2). In contrast, IDR-1018+M2 differentiated macrophages presented a chemokine profile similar to that of M2 macrophages.

### Differentiation of macrophages in the presence of IDR-1018 induced the expression of wound healing associated genes

**Figure 3:**
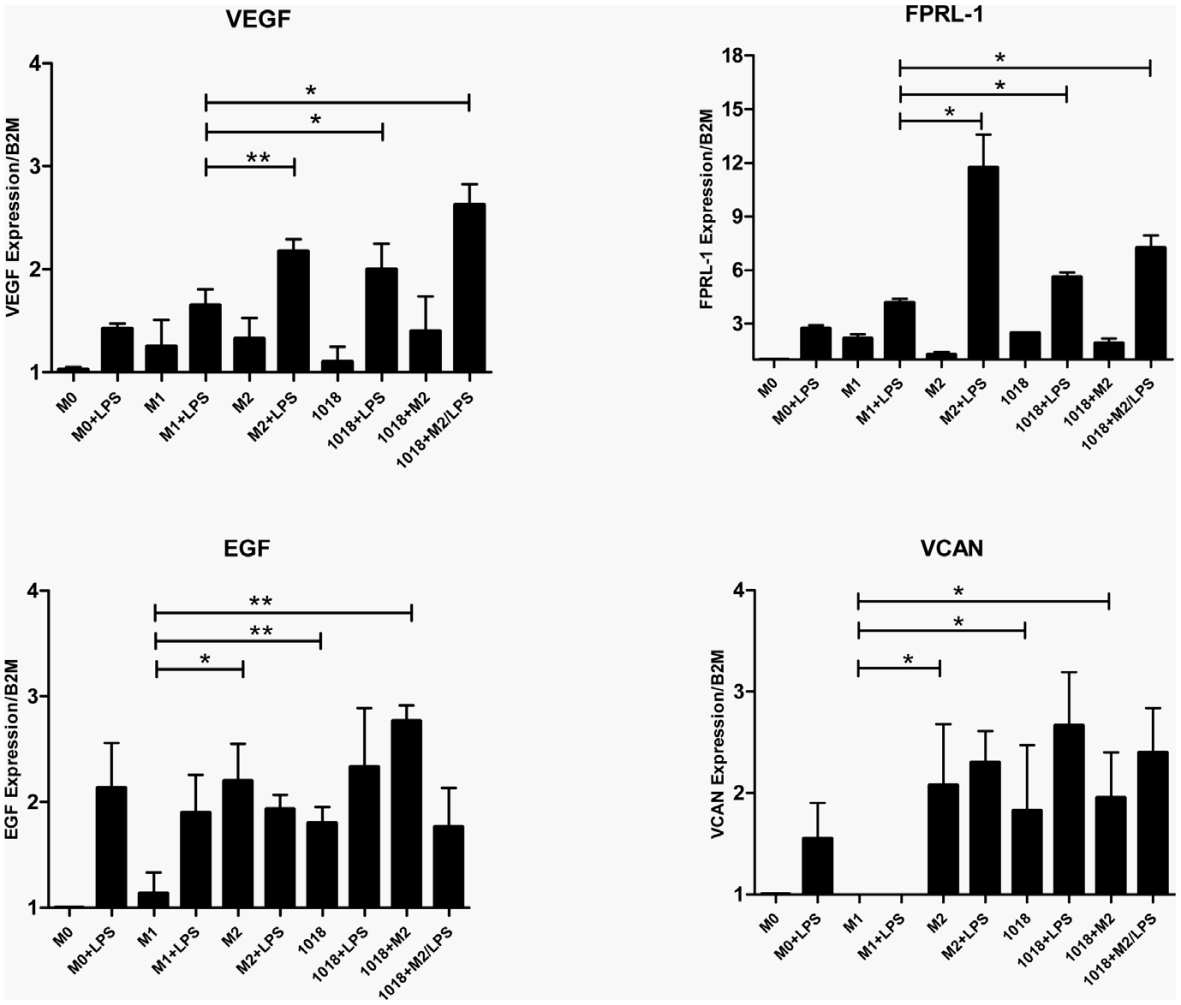
Differentiation of macrophages in the presence of IDR-1018 induced the expression of wound healing associated genes. Adherent monocytic cells were differentiated into macrophages in the presence of IFNγ (M1), M-CSF (M2), IDR-1018 alone or in combination with M-CSF (1018+M2), or left untreated (M0). These macrophages were then stimulated ± LPS. Four hours post-stimulation, transcriptional changes in wound healing associated genes were measured by RT-qPCR, and analyzed for significant differences when compared to the M1 phenotype. Mean ± SD results are presented and are representative of 4 biological replicates. **, P<0.01; *, P<0.05.

Wound healing is a characteristic function of M2 macrophages [32] and a number of wound-healing associated genes such as growth factors and components of the extracellular matrix are expressed in these cells. We recently demonstrated that IDR-1018 promotes wound healing in mice and pigs [33]. Therefore, the expression levels of these factors were investigated in IDR-1018 and IDR-1018+M2 differentiated macrophages (Figure 3). IDR-1018 and IDR-1018+M2 differentiated macrophages demonstrated differential effects on the expression of endothelial growth factor (EGF) and the proteoglycan Versican (VCAN) that were similar to or higher than those found on M2 macrophages. Although no significant differences were found in the basal levels of other wound healing genes such as vascular endothelial growth factor (VEGF) and formyl peptide receptor like-1 (FPRL-1), when IDR-1018 and IDR-1018+M2 macrophages were stimulated with LPS, major differential changes were observed, leading to a profile that resembled that of the LPS stimulated M2 control. This response to LPS was observed with 3 of the 4 genes but not with EGF.

### Macrophages differentiated in the presence of IDR-1018 displayed enhanced phagocytic properties towards apoptotic cells

Phagocytosis of apoptotic cells is another distinctive function of alternatively activated macrophages [34]. Using UV-induced apoptotic Jurkat cells labeled with CFDA-SE as targets for macrophages differentiated under different conditions, a phagocytosis assay was performed and analyzed by flow cytometry. Zebra plots were created for each treatment, showing the percentage of macrophages with CFDA-SE positive apoptotic jurkat cells (Figure 3A–S). The geometric mean was measured for positive CFDA-SE gated macrophages (Figure 3B–S). The phagocytic activity, assessed by the geometric mean of control M1 macrophages was similar to that of M0 cells while M2 cells demonstrated approximately twice as much phagocytosis. IDR-1018-differentiated cells demonstrated a significant but slight increase in phagocytosis compared to the M1 control, but it was still far less than the M2 control. Interestingly, the phagocytic activity of IDR-1018+M2 differentiated macrophages was greater than that of the M2 macrophages.

### IDR-1018- macrophages maintained plasticity enabling return to a pro-inflammatory state

**Figure 4:**
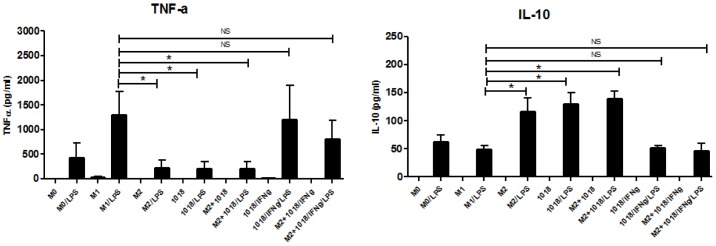
IDR-1018 differentiated macrophages maintained plasticity as they could return to a pro-inflammatory state. Macrophages were differentiated as described previously and treated ± LPS. In addition, macrophages differentiated in the presence of IDR-1018 alone or in combination with M-CSF, were subsequently treated with M1-inducing IFNγ for 24 hours then challenged with LPS. Four hours post-challenge, cytokine responses were analyzed by ELISA. Mean ± SD results are presented and are representative of 3 biological replicates. *, P<0.05.

Some of the pathologies associated with immunosuppression, e.g. endotoxin tolerance, are thought to result from macrophages that became locked into an M2 phenotype, indicating a loss of plasticity [18], [35]. Given the proposed use of IDR peptides as therapeutics, it was important to determine if differentiation in the presence of IDR-1018 affected the normal plasticity of macrophages and whether they were able to enter an M1 (LPS responsive) state. Therefore macrophages were differentiated as described previously, in the presence of IDR-1018 alone or in combination with M-CSF, and subsequently treated with the M1-promoting cytokine IFNγ. The pro-inflammatory cytokine TNF-α and the anti-inflammatory cytokine IL-10, as analyzed by ELISA, were used as phenotypic markers. IDR-1018 and IDR-1018+M2 differentiated macrophages stimulated with IFN-γ, exhibited increased production of TNF-α and reduced IL-10, similar to M1 macrophages, in contrast to the equivalent macrophages that had not been stimulated with IFN-γ, which demonstrated cytokine expression profiles partly resembling those of M2 macrophages (Figure 4). These results suggest that IDR-1018 differentiated macrophages maintain plasticity, allowing modulation of their responsiveness.

### IDR-1018 treated monocytes and monocyte-derived macrophages expressed transcription factors important for the development of M2 macrophages

**Figure 5:**
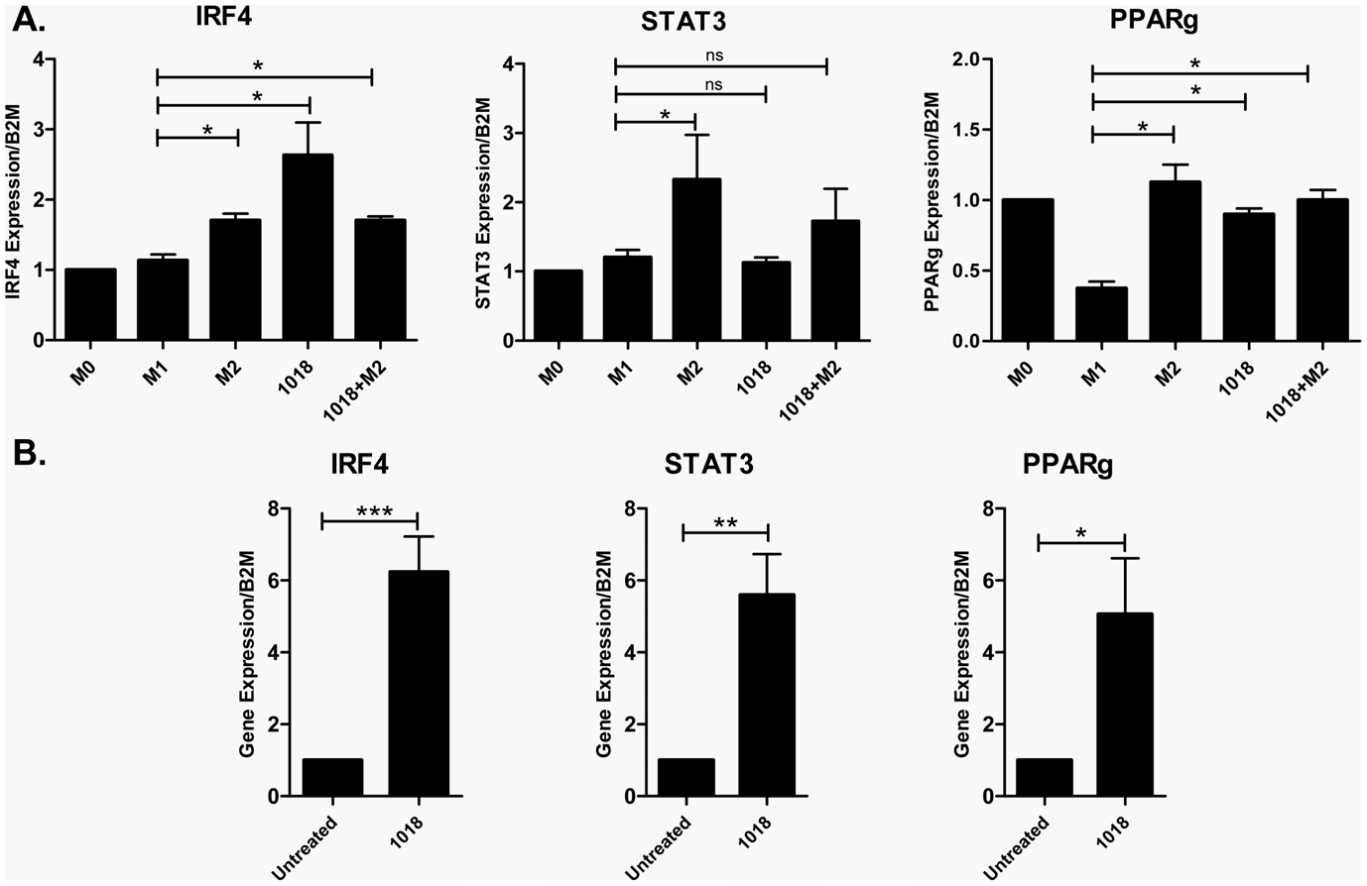
IDR-1018 treated monocytes and IDR-1018 differentiated macrophages expressed transcription factors important for the development of alternative (M2) macrophages. (A) Macrophages were differentiated in the presence of IDR-1018 or the combination of IDR-1018 and M2-inducing factor M-CSF, and RT-qPCR was performed to analyze the expression of different M2 specific transcription factors. (B) Additionally, monocytes were stimulated with IDR-1018 (5 ug/ml) for 4 hours. In both cases, RNA was isolated and the expression of transcription factors was analyzed by RT-qPCR. Mean ± SD results are presented and are representative of 3 biological replicates. *, P<0.05.

Several transcription factors that promote the development of M2 macrophages have been previously identified [16], [36], [37], [38]. Therefore we sought to analyze the role of these factors in IDR-1018 differentiated macrophages and IDR-1018 stimulated monocytes using RT-qPCR. Intermediate responses were observed for the 3 transcription factors analyzed: Interferon regulatory factor 4 (IRF4), signal transducer and activator of transcription 3 (STAT3) and peroxisome activated receptor gamma (PPARγ) (Figure 5A). IRF4 was not induced in M1 cells but was significantly upregulated in M2 cells. IDR-1018 differentiated macrophages demonstrated a high IRF4 induction while IDR-1018+M2 differentiated cells more resembled M2 cells. With respect to STAT3, IDR-1018 differentiated macrophages demonstrated no induction, like M1 cells, while IDR-1018+M2 differentiated cells, demonstrated a slight induction of this transcription factor. PPARγ was downregulated in M1 macrophages but not in M2 macrophages or macrophages differentiated in the presence of IDR-1018 or 1018+M2.

Interestingly, the expression of all three M2-promoting transcription factors, PPARγ, IRF4 and STAT3, was upregulated by 5- to 6-fold in monocytes stimulated with IDR-1018 compared to unstimulated monocytes (Figure 5B). Since IRF4 was hyperexpressed in IDR-1018 differentiated macrophages, we investigated the impact of its expression on downstream responses. High-throughput RNA-seq was performed on IDR-1018 differentiated monocytes, versus unstimulated monocytes, demonstrating 542 upregulated and 334 down regulated genes (table S2). Intersection of the 876 differentially expressed (DE) genes in IDR-1018 treated monocytes with M2-polarized macrophage microarray data revealed that 165 (19%) DE genes were also associated with M2 macrophage phenotype [11]. To determine the extent of IRF4 influence on the M2-subset in the IDR-1018 response, we examine the presence of IRF4 binding sites [23], and revealed 71 genes (41%) had previously demonstrated IRF4 binding (Table S3). Some of the genes identified are known to be associated with the development of M2 macrophages and/or other activities associated with the M2 phenotype including the mannose receptor (MR), PPARγ and matrix metalloprotease 9 (MMP9) [36], [39], [40].

## Discussion

The steady rise in antimicrobial resistance in recent years combined with a declining rate of antibiotic discovery has generated a major health challenge. Host-directed immunomodulatory therapies such as innate defence regulators (IDRs) represent a promising new approach to combat this problem [2]. A characteristic of IDR peptides, and natural host defence peptides like LL-37, is their ability to suppress pro-inflammatory responses induced by bacterial molecules like lipopolysaccharide (LPS), which led us to an initial hypothesis that these peptides might bias macrophages towards an M2 phenotype. Here, we demonstrated the ability of IDR-1018 to modulate the differentiation of macrophages, a cell type that plays a major role during the immune response towards infection and is critical to IDR anti-infective activity [41], [42]. However, contrary to our initial expectations, IDR-1018, when present during differentiation, led to a unique intermediate macrophage phenotype with different characteristics reflecting both the classical M1 and the alternative M2 phenotype. In contrast, differentiation of macrophages in the presence of IDR-1018 together with an M2-inducing factor M-CSF resulted in an apparently enhanced M2 phenotype.

The distinctive intermediate state promoted by IDR-1018, was characterized by the selective down-regulation of certain pro-inflammatory mediators associated with M1 macrophages [31], [43]. Thus TNF-α, COX-2 and IP-10 were substantially diminished in the IDR-1018 differentiated macrophages, while IL-12 and CCL-3 were only slightly reduced. Interestingly, although IDR-1018 differentiated macrophages exhibited substantial up-regulation of two anti-inflammatory mediators strongly associated with M2 macrophages, IL-10 and CCL-22, and the expression of others such as TGFβ remained unchanged.

Wound healing and phagocytosis of apoptotic cells are hallmark functions of M2 macrophages [8], [34], [44]. IDR-1018 [33], like natural host defence peptides, has been shown to promote wound healing in mice and pigs [45], [46]. Consistent with this, we observed here that IDR-1018 and IDR1018−M2 differentiated macrophages exhibited a basal increase in the expression of different wound healing genes (EGF, VCAN) that are critical for the process of wound healing and tissue repair. Interestingly, we found that certain wound healing genes such as VEGF and FPRL-1 were only differentially expressed after LPS stimulation, which may indicate that although these macrophages had developed an M2-like phenotype, they only expressed certain wound healing associated molecules in response to specific changes in the microenvironment. Phagocytosis of apoptotic cells was also affected by differentiation with IDR-1018 but only to a modest extent. In contrast, when IDR-1018 was used in combination with M-CSF, the phagocytic activity was even greater than that observed for M2 macrophages. These data suggest that IDR-1018 differentiated macrophages might play a major role during the resolution of an infection or after tissue injury by clearing the affected site of debris and apoptotic cells, a process required for the return to tissue homeostasis.

Macrophages display considerable plasticity, allowing them to change their responses depending on the challenges to which they are exposed. However, under certain circumstances, they may become locked in a specific state such as a classical M1 state, during chronic inflammatory and autoimmune diseases, or an alternative M2 state, during immunosuppressive disorders [35], [47]. If IDR-1018 caused macrophages to become locked into a particular state, and particularly an M2-like immunosuppressive state, this would represent a substantial limitation on its development as a therapeutic. Therefore we tested whether IDR-1018 influenced the plasticity of macrophages. Critically, both IDR-1018 differentiated and IDR-1018+M2 differentiated macrophages were able to alter their responses to a more M1-like phenotype after exposure to the M1 inducing cytokine IFNγ, demonstrating that the phenotype induced by IDR-1018 could indeed be reversed.

The molecular determinants of macrophage differentiation vary depending on the inducing factor. Since IDR-1018 appeared to promote an intermediate phenotype, with certain features of the M2 phenotype, we examined transcription factors known to be involved in the early development of this state. RT-qPCR analysis of monocytes treated with IDR-1018 for a short period of time (representing the early stages of differentiation) demonstrated the strong transcriptional upregulation of important transcription factors such as PPARγ, STAT3 and IRF4. In contrast, in later-stage IDR-1018 differentiated macrophages, only IRF4 remained up-regulated. This is partly consistent with studies done by Bohuel et al [36], who demonstrated that PPARγ is important to skewing mononuclear cells towards an M2 phenotype. Thus while several transcription factors, including PPARγ, are important for the initiation of differentiation in monocytes by IDR-1018, IRF4 might play an important role in sustaining the IDR-1018 phenotype once macrophages have matured and differentiated. Indeed IRF4 was found to be a central factor for the development of the M2 phenotype induced by M-CSF [16]. To further examine the role of IRF4 in macrophage differentiation induced by IDR-1018, we utilized system biology approaches by obtaining RNA-Seq data of IDR-1018 treated monocytes and integrated it with IRF4 binding site data from the literature [23]. Our analysis showed that 41% of the differentially expressed genes were common to M2 transcriptional data and contained an IRF4 binding sites, demonstrating the likelihood that these genes were controlled by the transcription factor IRF4. Importantly, many of the genes with IRF4 binding sites including PPARγ, mannose receptor (MR), and metallomieloperoxidase 9 (MMP9), have been associated with the development and key functions of the M2 phenotype

Based on the results presented here, we propose that the intermediate phenotype generated by IDR-1018, makes it a good candidate for modulating inflammatory disorders such as sepsis. During mid to later sage sepsis, there is an imbalance towards an immunosuppressive state, also known as endotoxin tolerance and recently recognized as being associated with the presence of M2-like mononuclear cells [10]. Endotoxin tolerance needs to be very carefully and modestly adjusted, since the complete abolition of this tolerant state would result in uncontrolled inflammation, while its enhancement could result in secondary infections. The ability of IDR-1018 to subtly modulate macrophage differentiation characterized by promoting anti-inflammatory activity with production of selected pro-inflammatory mediators, especially particular chemokines, makes it an attractive therapeutic option for this disorder. Additionally, IDR-1018, when used in combination with M-CSF, enhances the M2 regulatory phenotype. This response could be beneficial in pathologies associated with excessive inflammation such as the very early stages of sepsis, where the presence of a cytokine storm leads to a rapid organ dysfunction and eventually death. In fact, we recently demonstrated that IDR-1018 used in combination with anti-malaria treatment was able to alleviate cases of severe (cerebral) malaria, through suppression of life-threatening neural inflammation, as well as resolve severe invasive *Staphylococcus aureus* infections [5]. We propose that the observed IDR-1018 effects on macrophage differentiation represent one of the biological mechanisms underlying the success observed in that study, promoting dampening of proinflammatory responses while maintaining protective responses. Thus IDR-1018 used alone or in combination with other molecules provides an interesting alternative to traditional therapies, modulating the activity of immune cells such as macrophages to generate an appropriate protective response.

## Supporting Information

Figure S1 **Chemokine expression in PBMC after treatment with IDR-1018 and negative control peptides 1020 and 1015.** PBMC were treated with different peptide concentrations as shown in the graph. Twenty four hours post treatment, supernatants were collected and chemokine expression was analyzed by ELISA.
(TIF)

Figure S2 **Cytokine and chemokine responses of Macrophages differentiated in the presence of IDR-1018 and LL-37.** Adherent monocytic cells were differentiated into macrophages in the presence of MCSF for 7 days. IFNγ (M1), IL-4 (M2), IDR-1018 or IL-37, were added or left untreated (M0). Macrophages were then challenge with/without LPS for 4 hours after which, the cytokine and chemokine responses were measured by ELISA. The data was analyzed for significant differences between the treatments and the M1 phenotype. Mean ± SD results are presented and are representative of 4 biological replicates. **, P<0.01; *, P<0.05. Note that the IDR-1018 treatment described here, is equivalent to IDR-1018+M2 treatment used in the whole manuscript.
(TIF)

Figure S3 **Macrophages differentiated in the presence of IDR-1018 displayed enhanced phagocytic properties towards apoptotic cells.** Macrophages were differentiated in the presence of IFN-γ (M1), M-CSF (M2), IDR-1018 alone (1018) or in combination with M-CSF (M2+1018), or left untreated (M0). Then,macrophages were incubated for 4 hours with CFDA-SE labeled UV-induced apoptotic Jurkat cells. Macrophages were harvested and phogocytosis analyzed by flow cytometry, gating on the macrophage population. Representative zebra plots were created for each treatment, showing the percentage of macrophages with CFDA-SE positive apoptotic Jurkat cells (A). The geometric mean was measured for CFDA-SE positive gated macrophages (B). Mean ± SD results are presented and are representative of 3 biological replicates. ***, P<.0.0001; **, P<0.01; *, P<0.05.
(TIF)

Table S1 **Primer List.**
(DOCX)

Table S2 **Differentially expressed genes in IDR-1018 treated monocytes.** Human monocytes stimulated for 4 hours with 20 µg/ml IDR-1018, compared to unstimulated monocytes.
(DOCX)

Table S3 **M2 subset of IDR-1018 transcriptional data integrated with IRF-4 binding sites.** M2-phenotype associated genes containing IRF-4 binding sites that were differentially expressed in human monocytes stimulated with 20 µg/ml IDR-1018. Genes highlighted/undelined demonstrated IRF4 binding within the annotated gene's structure.
(DOCX)
