# Supplementary figures and images for: Synthetic Cationic Peptide IDR-1018 Modulates Human Macrophage Differentiation
Source: PLoS One. 2013 Jan 7;8(1):e52449. doi: 10.1371/journal.pone.0052449 (PMC3538731; doi:10.1371/journal.pone.0052449)

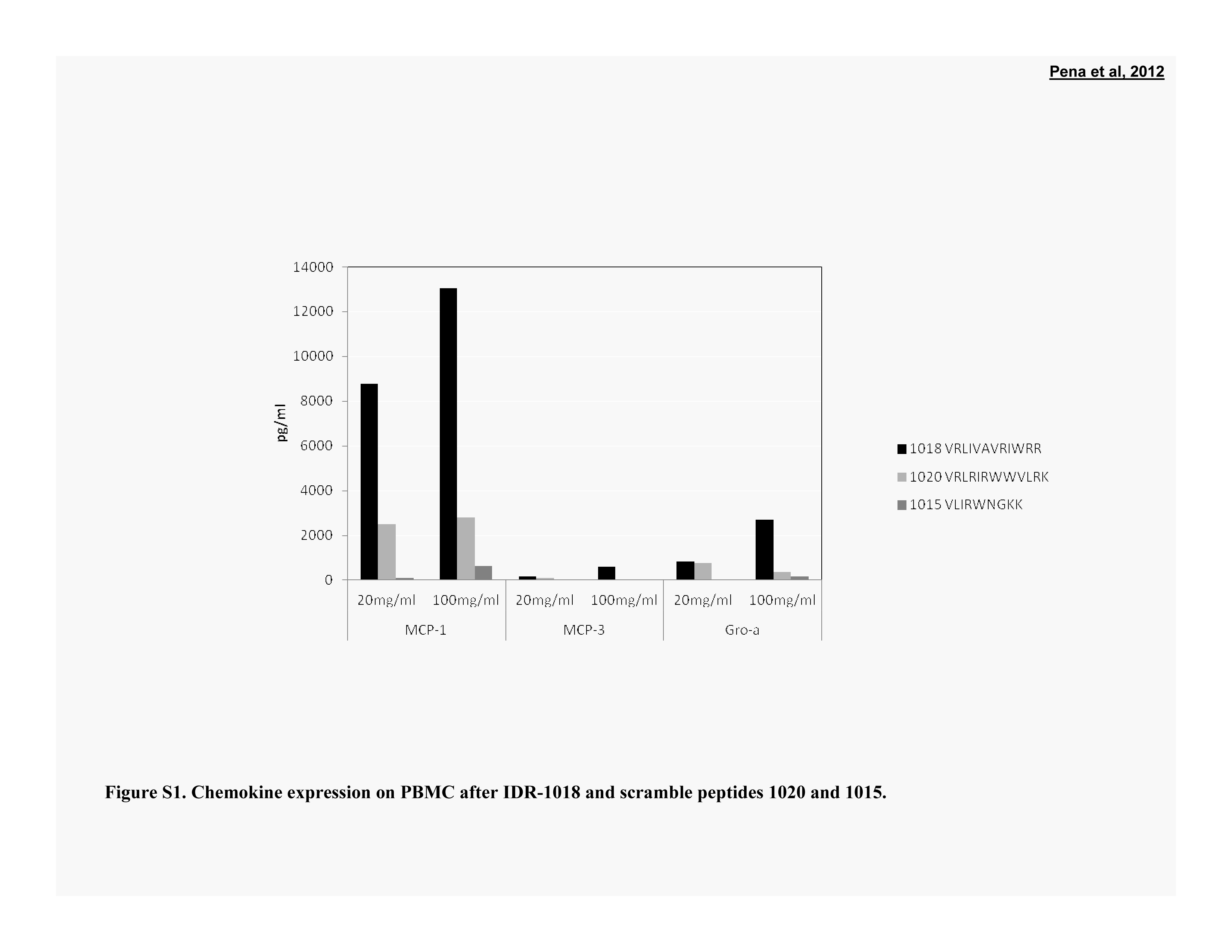

Supplement: Figure S1 — Chemokine expression in PBMC after treatment with IDR-1018 and negative control peptides 1020 and 1015. PBMC were treated with different peptide concentrations as shown in the graph. Twenty four hours post treatment, supernatants were collected and chemokine expression was analyzed by ELISA. (TIF) [file pone.0052449.s001.tif]

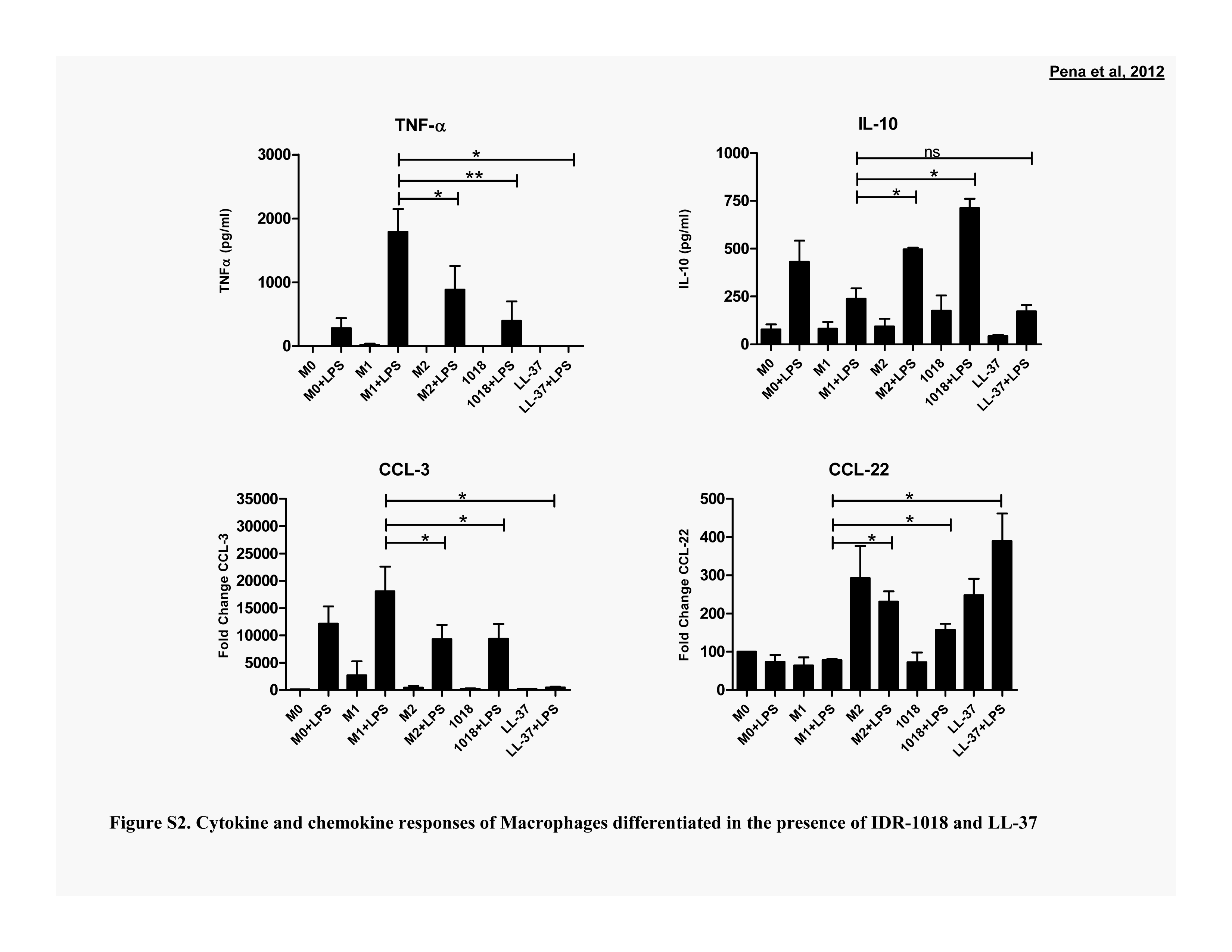

Supplement: Figure S2 — Cytokine and chemokine responses of Macrophages differentiated in the presence of IDR-1018 and LL-37. Adherent monocytic cells were differentiated into macrophages in the presence of MCSF for 7 days. IFNγ (M1), IL-4 (M2), IDR-1018 or IL-37, were added or left untreated (M0). Macrophages were then challenge with/without LPS for 4 hours after which, the cytokine and chemokine responses were measured by ELISA. The data was analyzed for significant differences between the treatments and the M1 phenotype. Mean ± SD results are presented and are representative of 4 biological replicates. **, P<0.01; *, P<0.05. Note that the IDR-1018 treatment described here, is equivalent to IDR-1018+M2 treatment used in the whole manuscript. (TIF) [file pone.0052449.s002.tif]

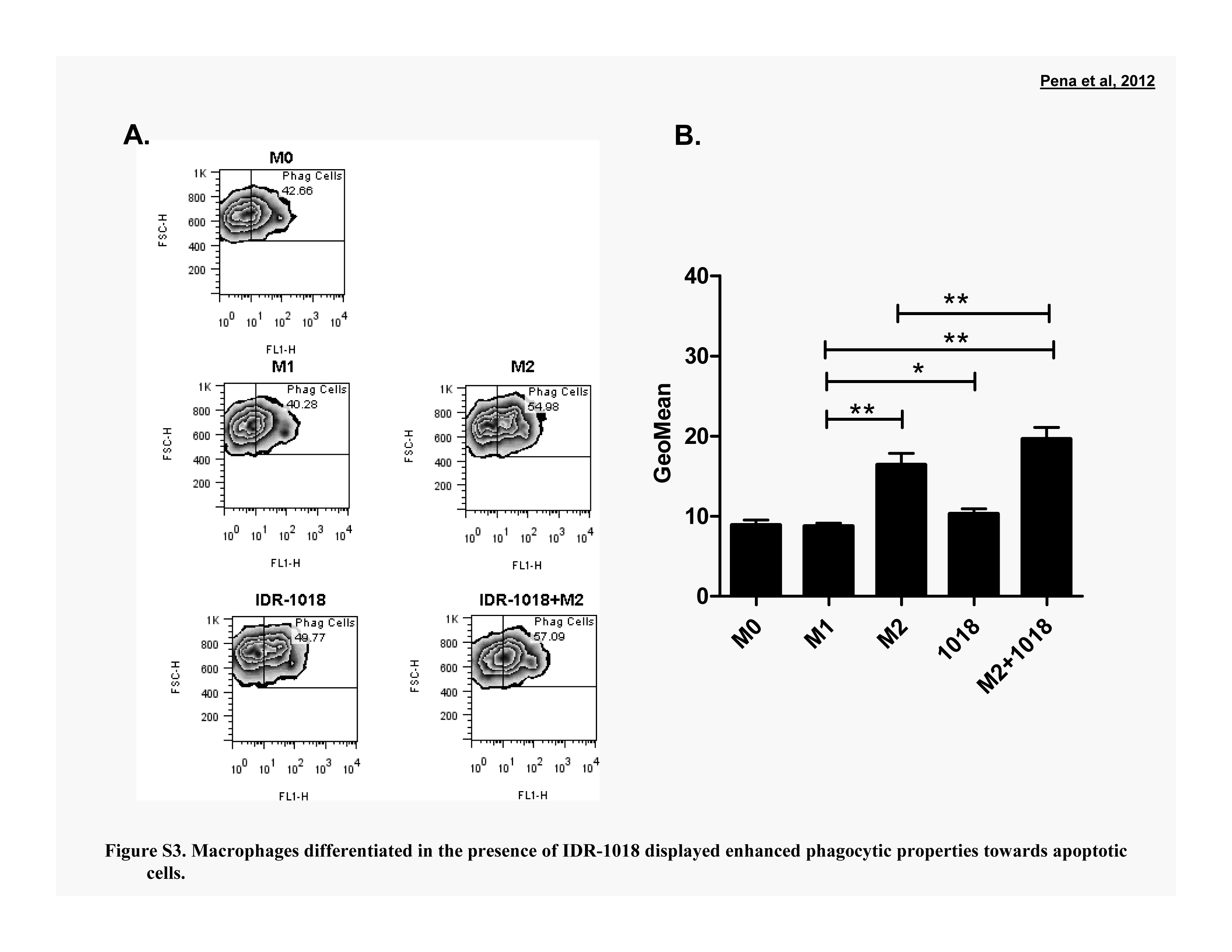

Supplement: Figure S3 — Macrophages differentiated in the presence of IDR-1018 displayed enhanced phagocytic properties towards apoptotic cells. Macrophages were differentiated in the presence of IFN-γ (M1), M-CSF (M2), IDR-1018 alone (1018) or in combination with M-CSF (M2+1018), or left untreated (M0). Then,macrophages were incubated for 4 hours with CFDA-SE labeled UV-induced apoptotic Jurkat cells. Macrophages were harvested and phogocytosis analyzed by flow cytometry, gating on the macrophage population. Representative zebra plots were created for each treatment, showing the percentage of macrophages with CFDA-SE positive apoptotic Jurkat cells (A). The geometric mean was measured for CFDA-SE positive gated macrophages (B). Mean ± SD results are presented and are representative of 3 biological replicates. ***, P<.0.0001; **, P<0.01; *, P<0.05. (TIF) [file pone.0052449.s003.tif]
