# Supplementary material for: Synthetic Cationic Peptide IDR-1018 Modulates Human Macrophage Differentiation
Source: PLoS One. 2013 Jan 7;8(1):e52449. doi: 10.1371/journal.pone.0052449 (PMC3538731; doi:10.1371/journal.pone.0052449)
Supplement: Table S1 — Primer List. (DOCX) [file pone.0052449.s004.docx]

**Table S1. Primer List**

| **Gene** | **Forward** | **Reverse** |
| --- | --- | --- |
| Cox-2 | GTTCCACCCGCAGTACAG | GGAGCGGGAAGAACTTGC |
| IL12-p40 | CGGTCATCTGCCGCAAA | TGCCCATTCGCTCCAAGA |
| IL12-p35 | GGTGAAGGCATGGGAACATT | TGCCCATTCGCTCCAAGA |
| VEGF | GCACCATGGCAGAAGG | CTCGATTGGATGGCAGTACT |
| EGF | ACGCCCTAAGTCGAGACCGGA | TCGGGTGAGGAACAACCGCT |
| Versican | GTGACTATGGCTGGCACAAATTCC | GGTTGGGTCTCCAATTCTCGTATTGC |
| IRF-4 | TCCCCACAGAGCCAAGCATAAGGT | AGGGAGCGGCCGTGGTGAGCA |
| STAT-3 | CCTTGGCTGGCTAGCTCG | TGAGTTGCCAAATCCGGC |
| PPARγ | AGTCCTCACAGCTGTTTGCCAAGC | GAGCGGGTGAAGACTCATGTCTGTC |
