# Supplementary material for: Synthetic Cationic Peptide IDR-1018 Modulates Human Macrophage Differentiation
Source: PLoS One. 2013 Jan 7;8(1):e52449. doi: 10.1371/journal.pone.0052449 (PMC3538731; doi:10.1371/journal.pone.0052449)
Supplement: Table S2 — Differentially expressed genes in IDR-1018 treated monocytes. Human monocytes stimulated for 4 hours with 20 µg/ml IDR-1018, compared to unstimulated monocytes. (DOCX) [file pone.0052449.s005.docx]

**Table S2: Differentially expressed genes in IDR-1018 treated monocytes.** Human monocytes stimulated for 4 hours with 20 μg/ml IDR-1018, compared to unstimulated monocytes.

| **ENTREZ GENE ID** | **DESCRIPTION** | **Fold change IDB** | **P Value** |
| --- | --- | --- | --- |
| 140458 | ASB5 [ankyrin repeat and SOCS box-containing 5] | 18.70 | 2.67E-06 |
| 7015 | TERT [telomerase reverse transcriptase] | 17.80 | 4.35E-04 |
| 202500 | TCTE1 [t-complex-associated-testis-expressed 1] | 16.50 | 1.09E-03 |
| 347148 | QRFP [pyroglutamylated RFamide peptide] | 16.10 | 1.09E-03 |
| 727910 | TLCD2 [TLC domain containing 2] | 16.00 | 1.62E-05 |
| 123970 | C16orf78 [chromosome 16 open reading frame 78] | 15.40 | 1.74E-03 |
| 7101 | NR2E1 [nuclear receptor subfamily 2, group E, member 1] | 15.00 | 1.74E-03 |
| 6005 | RHAG [Rh-associated glycoprotein] | 13.30 | 7.37E-05 |
| 170063 | CXorf22 [chromosome X open reading frame 22] | 13.30 | 4.52E-03 |
| 283985 | FADS6 [fatty acid desaturase domain family, member 6] | 13.20 | 4.52E-03 |
| 443 | ASPA [aspartoacylase] | 12.20 | 7.34E-03 |
| 58530 | LY6G6D [lymphocyte antigen 6 complex, locus G6D] | 12.10 | 7.34E-03 |
| 84647 | PLA2G12B [phospholipase A2, group XIIB] | 12.00 | 7.34E-03 |
| 11095 | ADAMTS8 [metallopeptidase] | 11.10 | 3.58E-04 |
| 4222 | MEOX1 [mesenchyme homeobox 1] | 11.00 | 1.20E-02 |
| 416 | ARSF [arylsulfatase F] | 11.00 | 5.37E-04 |
| 112802 | KRT71 [keratin 71] | 11.00 | 1.20E-02 |
| 23732 | C9orf4 [chromosome 9 open reading frame 4] | 10.80 | 3.29E-05 |
| 6696 | SPP1 [secreted phosphoprotein 1] | 10.10 | 5.34E-16 |
| 57481 | KIAA1210 [KIAA1210] | 9.95 | 1.97E-02 |
| 148170 | CDC42EP5 [CDC42 effector 5, Rho GTPase binding] | 9.94 | 2.35E-05 |
| 440097 | DBX2 [developing brain homeobox 2] | 9.94 | 1.97E-02 |
| 10332 | CLEC4M [C-type lectin domain family 4, member M] | 9.92 | 1.97E-02 |
| 285126 | DNAJC5G [DnaJ/Hsp40 homolog] | 9.81 | 4.84E-05 |
| 2670 | GFAP [glial fibrillary acidic protein] | 9.62 | 1.35E-04 |
| 221223 | CES7 [carboxylesterase 7] | 9.30 | 1.93E-04 |
| 3171 | FOXA3 [forkhead box A3] | 8.96 | 5.63E-07 |
| 10253 | SPRY2 [sprouty homolog 2 (Drosophila)] | 8.83 | 8.70E-10 |
| 401934 | LOC401934 [hypothetical LOC401934] | 8.81 | 3.24E-02 |
| 9843 | HEPH [hephaestin] | 8.80 | 3.24E-02 |
| 84249 | PSD2 [pleckstrin and Sec7 domain containing 2] | 8.78 | 3.24E-02 |
| 114898 | C1QTNF2 [C1q and tumor necrosis factor related protein 2] | 8.78 | 3.24E-02 |
| 57030 | SLC17A7 [solute carrier family 17, member 7] | 8.71 | 2.83E-03 |
| 8419 | BFSP2 [beaded filament structural protein 2, phakinin] | 8.49 | 8.56E-06 |
| 285315 | C3orf33 [chromosome 3 open reading frame 33] | 7.92 | 9.03E-08 |
| 389852 | SPACA5 [sperm acrosome associated 5] | 7.83 | 6.63E-03 |
| 729201 | SPACA5B [sperm acrosome associated 5B] | 7.83 | 6.63E-03 |
| 149428 | BNIPL [BCL2 E1B 19kD interacting protein like] | 7.69 | 2.98E-04 |
| 29943 | PADI1 [peptidyl arginine deiminase, type I] | 7.67 | 6.63E-03 |
| 127003 | C1orf194 [chromosome 1 open reading frame 194] | 7.65 | 1.02E-02 |
| 730249 | IRG1 [immunoresponsive 1 homolog (mouse)] | 7.61 | 1.59E-10 |
| 54443 | ANLN [anillin, actin binding protein] | 7.57 | 9.43E-05 |
| 284573 | C1orf157 [chromosome 1 open reading frame 157] | 7.47 | 4.18E-04 |
| 6364 | CCL20 [chemokine (C-C motif) ligand 20] | 7.45 | 5.99E-07 |
| 386617 | KCTD8 [potassium channel tetramerisation domain containing 8] | 7.27 | 1.23E-05 |
| 794 | CALB2 [calbindin 2] | 7.19 | 1.02E-02 |
| 64388 | GREM2 [gremlin 2] | 7.18 | 1.32E-09 |
| 118932 | ANKRD22 [ankyrin repeat domain 22] | 7.02 | 3.67E-10 |
| 57484 | RNF150 [ring finger protein 150] | 6.99 | 1.93E-04 |
| 116071 | BATF2 [basic leucine zipper transcription factor, ATF-like 2] | 6.98 | 5.40E-09 |
| 84929 | FIBCD1 [fibrinogen C domain containing 1] | 6.91 | 3.85E-03 |
| 4283 | CXCL9 [chemokine (C-X-C motif) ligand 9] | 6.85 | 6.03E-09 |
| 3627 | CXCL10 [chemokine (C-X-C motif) ligand 10] | 6.78 | 1.23E-11 |
| 339501 | PRSS38 [protease, serine, 38] | 6.59 | 4.52E-05 |
| 200132 | TCTEX1D1 [Tctex1 domain containing 1] | 6.54 | 3.85E-03 |
| 6273 | S100A2 [S100 calcium binding protein A2] | 6.51 | 2.80E-09 |
| 1610 | DAO [D-amino-acid oxidase] | 6.48 | 1.71E-04 |
| 2687 | GGT5 [gamma-glutamyltransferase 5] | 6.37 | 2.00E-05 |
| 8200 | GDF5 [growth differentiation factor 5] | 6.36 | 4.19E-04 |
| 340654 | LIPM [lipase, family member M] | 6.23 | 5.66E-03 |
| 146439 | CCDC64B [coiled-coil domain containing 64B] | 6.20 | 5.66E-03 |
| 222950 | C7orf51 [chromosome 7 open reading frame 51] | 6.14 | 6.27E-05 |
| 93649 | MYOCD [myocardin] | 6.10 | 2.43E-02 |
| 344022 | NOTO [notochord homeobox] | 6.09 | 2.43E-02 |
| 344657 | LRRIQ4 [leucine-rich repeats and IQ motif containing 4] | 6.08 | 2.43E-02 |
| 10692 | RRH [retinal pigment epithelium-derived rhodopsin homolog] | 6.08 | 2.43E-02 |
| 137362 | GOT1L1 [glutamic-oxaloacetic transaminase 1-like 1] | 6.01 | 2.43E-02 |
| 494514 | C18orf56 [chromosome 18 open reading frame 56] | 6.01 | 1.66E-03 |
| 29126 | CD274 [CD274 molecule] | 5.99 | 3.03E-09 |
| 419 | ART3 [ADP-ribosyltransferase 3] | 5.92 | 1.76E-10 |
| 1594 | CYP27B1 [cytochrome P450, family 27 B, polypeptide 1] | 5.86 | 8.33E-03 |
| 83650 | AMAC1L2 [acyl-malonyl condensing enzyme 1-like 2] | 5.56 | 2.08E-03 |
| 8431 | NR0B2 [nuclear receptor subfamily 0, group B, member 2] | 5.55 | 1.23E-02 |
| 359787 | DPPA3 [developmental pluripotency associated 3] | 5.53 | 2.08E-03 |
| 4059 | BCAM [basal cell adhesion molecule (Lutheran blood group)] | 5.48 | 5.52E-04 |
| 342527 | SMTNL2 [smoothelin-like 2] | 5.46 | 4.75E-03 |
| 338707 | B4GALNT4 [beta-1,4-N-acetyl-galactosaminyl transferase 4] | 5.38 | 1.00E-03 |
| 22997 | IGSF9B [immunoglobulin superfamily, member 9B] | 5.32 | 2.67E-06 |
| 10537 | UBD [ubiquitin D] | 5.28 | 2.88E-03 |
| 81492 | RSPH6A [radial spoke head 6 homolog A (Chlamydomonas)] | 5.27 | 2.93E-04 |
| 400165 | C13orf35 [chromosome 13 open reading frame 35] | 5.19 | 6.77E-03 |
| 1551 | CYP3A7 [cytochrome P450, family 3A, polypeptide 7] | 5.16 | 1.82E-02 |
| 4582 | MUC1 [mucin 1, cell surface associated] | 5.11 | 3.68E-06 |
| 389558 | FAM180A [family with sequence similarity 180, member A] | 5.08 | 1.82E-02 |
| 57159 | TRIM54 [tripartite motif-containing 54] | 5.05 | 4.96E-04 |
| 4146 | MATN1 [matrilin 1, cartilage matrix protein] | 5.00 | 5.58E-05 |
| 2119 | ETV5 [ets variant 5] | 4.92 | 1.08E-04 |
| 57758 | SCUBE2 [signal peptide, CUB domain, EGF-like 2] | 4.91 | 1.74E-04 |
| 2254 | FGF9 [fibroblast growth factor 9 (glia-activating factor)] | 4.85 | 5.54E-03 |
| 100131137 | BSPH1 [binder of sperm protein homolog 1] | 4.78 | 5.54E-03 |
| 3083 | HGFAC [HGF activator] | 4.75 | 2.68E-02 |
| 26974 | ZNF285 [zinc finger protein 285] | 4.72 | 8.71E-05 |
| 54798 | DCHS2 [dachsous 2 (Drosophila)] | 4.70 | 5.54E-03 |
| 221409 | SPATS1 [spermatogenesis associated, serine-rich 1] | 4.69 | 1.38E-02 |
| 1960 | EGR3 [early growth response 3] | 4.64 | 2.14E-08 |
| 113452 | TMEM54 [transmembrane protein 54] | 4.62 | 7.69E-03 |
| 57615 | ZNF492 [zinc finger protein 492] | 4.59 | 4.57E-03 |
| 3785 | KCNQ2 [potassium voltage-gated channel, KQT-like, member 2] | 4.57 | 4.39E-04 |
| 139135 | PASD1 [PAS domain containing 1] | 4.46 | 3.37E-03 |
| 1268 | CNR1 [cannabinoid receptor 1 (brain)] | 4.45 | 3.34E-03 |
| 26531 | OR11A1 [olfactory receptor, family 11, subfamily A, member 1] | 4.42 | 1.97E-02 |
| 2321 | FLT1 [fms-related tyrosine kinase 1] | 4.40 | 5.64E-08 |
| 56833 | SLAMF8 [SLAM family member 8] | 4.40 | 2.41E-06 |
| 8190 | MIA [melanoma inhibitory activity] | 4.39 | 3.96E-02 |
| 136263 | C7orf45 [chromosome 7 open reading frame 45] | 4.38 | 1.38E-02 |
| 138240 | C9orf57 [chromosome 9 open reading frame 57] | 4.36 | 7.69E-03 |
| 134526 | ACOT12 [acyl-CoA thioesterase 12] | 4.36 | 3.96E-02 |
| 374739 | TEPP [testis, prostate and placenta expressed] | 4.35 | 1.97E-02 |
| 114795 | TMEM132B [transmembrane protein 132B] | 4.34 | 1.97E-02 |
| 100289074 | LOC100289074 [hypothetical protein LOC100289074] | 4.34 | 1.38E-02 |
| 143903 | LAYN [layilin] | 4.34 | 1.07E-02 |
| 4131 | MAP1B [microtubule-associated protein 1B] | 4.32 | 1.83E-03 |
| 10570 | DPYSL4 [dihydropyrimidinase-like 4] | 4.25 | 6.86E-04 |
| 118611 | C10orf90 [chromosome 10 open reading frame 90] | 4.25 | 8.44E-03 |
| 130075 | OR9A4 [olfactory receptor, family 9, subfamily A, member 4] | 4.24 | 1.68E-04 |
| 9074 | CLDN6 [claudin 6] | 4.10 | 2.48E-03 |
| 9955 | HS3ST3A1 [heparan sulfate 3-O-sulfotransferase 3A1] | 4.08 | 6.51E-04 |
| 140766 | ADAMTS14 [ADAM metallopeptidase 14] | 4.03 | 3.89E-04 |
| 54566 | EPB41L4B [erythrocyte membrane protein band 4.1 like 4B] | 3.99 | 2.75E-03 |
| 5996 | RGS1 [regulator of G-protein signaling 1] | 3.97 | 1.04E-06 |
| 5881 | RAC3 [rho family, small GTP binding protein Rac3] | 3.96 | 2.07E-02 |
| 25907 | TMEM158 [transmembrane protein 158 (gene/pseudogene)] | 3.95 | 7.66E-07 |
| 6579 | SLCO1A2 [solute carrier organic anion transporter family, 1A2] | 3.90 | 4.02E-02 |
| 57526 | PCDH19 [protocadherin 19] | 3.89 | 4.02E-02 |
| 257044 | C1orf101 [chromosome 1 open reading frame 101] | 3.89 | 3.97E-04 |
| 2669 | GEM [GTP binding protein overexpressed in skeletal muscle] | 3.88 | 7.69E-03 |
| 114026 | ZIM3 [zinc finger, imprinted 3] | 3.88 | 6.78E-03 |
| 1088 | CEACAM8 [carcinoembryonic antigen-related cell adhesion 8] | 3.87 | 1.56E-02 |
| 27231 | ITGB1BP3 [integrin beta 1 binding protein 3] | 3.87 | 8.46E-04 |
| 399473 | SPRED3 [sprouty-related, EVH1 domain containing 3] | 3.86 | 4.02E-02 |
| 79822 | ARHGAP28 [Rho GTPase activating protein 28] | 3.78 | 4.02E-02 |
| 196500 | C12orf53 [chromosome 12 open reading frame 53] | 3.73 | 2.87E-02 |
| 64866 | CDCP1 [CUB domain containing protein 1] | 3.69 | 1.62E-06 |
| 51513 | ETV7 [ets variant 7] | 3.67 | 3.00E-04 |
| 6373 | CXCL11 [chemokine (C-X-C motif) ligand 11] | 3.63 | 2.09E-04 |
| 100289200 | LOC100289200 [similar to roundabout] | 3.61 | 1.63E-03 |
| 2633 | GBP1 [guanylate binding protein 1, interferon-inducible, 67kDa] | 3.61 | 1.55E-06 |
| 57569 | ARHGAP20 [Rho GTPase activating protein 20] | 3.61 | 2.68E-02 |
| 387700 | SLC16A12 [solute carrier family 16, member 12] | 3.59 | 1.21E-02 |
| 284677 | C1orf204 [chromosome 1 open reading frame 204] | 3.59 | 7.84E-05 |
| 391123 | VSIG8 [V-set and immunoglobulin domain containing 8] | 3.59 | 7.84E-05 |
| 145864 | HAPLN3 [hyaluronan and proteoglycan link protein 3] | 3.55 | 3.18E-05 |
| 2152 | F3 [coagulation factor III (thromboplastin, tissue factor)] | 3.53 | 4.46E-05 |
| 2538 | G6PC [glucose-6-phosphatase, catalytic subunit] | 3.52 | 1.49E-02 |
| 160762 | CCDC63 [coiled-coil domain containing 63] | 3.49 | 3.51E-03 |
| 9615 | GDA [guanine deaminase] | 3.45 | 5.54E-04 |
| 4915 | NTRK2 [neurotrophic tyrosine kinase, receptor, type 2] | 3.45 | 3.98E-02 |
| 64090 | GAL3ST2 [galactose-3-O-sulfotransferase 2] | 3.43 | 2.15E-04 |
| 23090 | ZNF423 [zinc finger protein 423] | 3.42 | 1.03E-03 |
| 2324 | FLT4 [fms-related tyrosine kinase 4] | 3.42 | 2.88E-02 |
| 5774 | PTPN3 [protein tyrosine phosphatase, non-receptor type 3] | 3.38 | 2.88E-02 |
| 11189 | CELF3 [CUGBP, Elav-like family member 3] | 3.32 | 3.96E-02 |
| 6474 | SHOX2 [short stature homeobox 2] | 3.32 | 5.93E-03 |
| 4609 | MYC [v-myc myelocytomatosis viral oncogene homolog (avian)] | 3.30 | 1.49E-05 |
| 643832 | GAGE10 [G antigen 10] | 3.29 | 2.88E-02 |
| 57116 | ZNF695 [zinc finger protein 695] | 3.29 | 8.62E-04 |
| 2209 | FCGR1A [Fc fragment of IgG, high affinity Ia, receptor (CD64)] | 3.28 | 1.29E-05 |
| 3960 | LGALS4 [lectin, galactoside-binding, soluble, 4] | 3.28 | 9.53E-03 |
| 117581 | TWIST2 [twist homolog 2 (Drosophila)] | 3.27 | 3.96E-02 |
| 6528 | SLC5A5 [solute carrier family 5, member 5] | 3.27 | 8.66E-04 |
| 2210 | FCGR1B [Fc fragment of IgG, high affinity Ib, receptor (CD64)] | 3.27 | 1.46E-05 |
| 50486 | G0S2 [G0/G1switch 2] | 3.24 | 1.12E-05 |
| 100289688 | LOC100289688 [similar to matrix metalloproteinase 19 isoform] | 3.21 | 1.33E-05 |
| 4327 | MMP19 [matrix metallopeptidase 19] | 3.21 | 1.33E-05 |
| 57530 | CGN [cingulin] | 3.20 | 7.67E-03 |
| 3552 | IL1A [interleukin 1, alpha] | 3.20 | 1.80E-05 |
| 9956 | HS3ST2 [heparan sulfate (glucosamine) 3-O-sulfotransferase 2] | 3.20 | 1.27E-03 |
| 2260 | FGFR1 [fibroblast growth factor receptor 1] | 3.20 | 1.94E-05 |
| 10693 | CCT6B [chaperonin containing TCP1, subunit 6B (zeta 2)] | 3.18 | 9.53E-03 |
| 728780 | ANKDD1B [ankyrin repeat and death domain containing 1B] | 3.17 | 5.93E-03 |
| 7293 | TNFRSF4 [tumor necrosis factor receptor superfamily, 4] | 3.16 | 7.79E-05 |
| 100288346 | LOC100288346 [hypothetical LOC100288346] | 3.15 | 8.55E-03 |
| 3741 | KCNA5 [potassium voltage-gated channel, member 5] | 3.12 | 1.28E-02 |
| 132954 | PDCL2 [phosducin-like 2] | 3.11 | 4.02E-02 |
| 653707 | LOC653707 [phosducin-like 2 pseudogene] | 3.11 | 4.02E-02 |
| 80832 | APOL4 [apolipoprotein L, 4] | 3.11 | 7.23E-05 |
| 401647 | GOLGA7B [golgin A7 family, member B] | 3.10 | 2.85E-02 |
| 23322 | RPGRIP1L [RPGRIP1-like] | 3.10 | 7.33E-04 |
| 140691 | TRIM69 [tripartite motif-containing 69] | 3.09 | 4.13E-03 |
| 3624 | INHBA [inhibin, beta A] | 3.08 | 6.83E-04 |
| 51129 | ANGPTL4 [angiopoietin-like 4] | 3.07 | 3.95E-04 |
| 9837 | GINS1 [GINS complex subunit 1 (Psf1 homolog)] | 3.06 | 1.42E-03 |
| 56246 | MRAP [melanocortin 2 receptor accessory protein] | 3.05 | 1.25E-02 |
| 482 | ATP1B2 [ATPase, Na+/K+ transporting, beta 2 polypeptide] | 3.05 | 2.13E-02 |
| 8645 | KCNK5 [potassium channel, subfamily K, member 5] | 3.03 | 1.31E-02 |
| 3553 | IL1B [interleukin 1, beta] | 3.02 | 2.84E-05 |
| 57817 | HAMP [hepcidin antimicrobial peptide] | 3.00 | 3.68E-02 |
| 8777 | MPDZ [multiple PDZ domain protein] | 3.00 | 2.12E-02 |
| 23187 | PHLDB1 [pleckstrin homology-like domain, family B, 1] | 2.99 | 4.53E-05 |
| 2919 | CXCL1 [chemokine (C-X-C motif) ligand 1] | 2.97 | 5.64E-05 |
| 282996 | RBM20 [RNA binding motif protein 20] | 2.95 | 5.20E-03 |
| 9242 | MSC [musculin] | 2.94 | 5.99E-05 |
| 85477 | SCIN [scinderin] | 2.92 | 1.10E-02 |
| 1846 | DUSP4 [dual specificity phosphatase 4] | 2.92 | 2.39E-04 |
| 11309 | SLCO2B1 [solute carrier organic anion transporter family, 2B1] | 2.92 | 2.14E-03 |
| 81501 | TM7SF4 [transmembrane 7 superfamily member 4] | 2.91 | 1.32E-03 |
| 245936 | DEFB123 [defensin, beta 123] | 2.91 | 4.02E-02 |
| 1959 | EGR2 [early growth response 2] | 2.91 | 5.83E-05 |
| 7474 | WNT5A [wingless-type MMTV integration site family, 5A] | 2.91 | 3.13E-04 |
| 91947 | ARRDC4 [arrestin domain containing 4] | 2.91 | 6.75E-05 |
| 10215 | OLIG2 [oligodendrocyte lineage transcription factor 2] | 2.88 | 1.30E-03 |
| 100288439 | LOC100288439 [hypothetical protein LOC100288439] | 2.88 | 2.14E-02 |
| 3659 | IRF1 [interferon regulatory factor 1] | 2.86 | 6.89E-05 |
| 1815 | DRD4 [dopamine receptor D4] | 2.86 | 2.30E-03 |
| 169044 | COL22A1 [collagen, type XXII, alpha 1] | 2.86 | 1.34E-02 |
| 2921 | CXCL3 [chemokine (C-X-C motif) ligand 3] | 2.86 | 7.95E-05 |
| 23114 | NFASC [neurofascin] | 2.84 | 4.81E-02 |
| 84911 | ZNF382 [zinc finger protein 382] | 2.83 | 2.05E-02 |
| 57007 | CXCR7 [chemokine (C-X-C motif) receptor 7] | 2.82 | 3.70E-04 |
| 89944 | GLB1L2 [galactosidase, beta 1-like 2] | 2.81 | 2.71E-02 |
| 100129603 | LOC100129603 [hypothetical LOC100129603] | 2.81 | 2.14E-02 |
| 441317 | FAM90A7 [family with sequence similarity 90, member A7] | 2.81 | 2.65E-02 |
| 113828 | FAM83F [family with sequence similarity 83, member F] | 2.80 | 4.59E-02 |
| 5891 | RAGE [renal tumor antigen] | 2.80 | 3.07E-03 |
| 5743 | PTGS2 [prostaglandin-endoperoxide synthase 2] | 2.79 | 1.17E-04 |
| 2335 | FN1 [fibronectin 1] | 2.79 | 1.03E-04 |
| 126147 | NTN5 [netrin 5] | 2.78 | 4.77E-03 |
| 92565 | FANK1 [fibronectin type III and ankyrin repeat domains 1] | 2.78 | 7.74E-03 |
| 85409 | NKD2 [naked cuticle homolog 2 (Drosophila)] | 2.77 | 2.14E-02 |
| 11199 | ANXA10 [annexin A10] | 2.77 | 2.59E-02 |
| 6236 | RRAD [Ras-related associated with diabetes] | 2.76 | 8.47E-04 |
| 29842 | TFCP2L1 [transcription factor CP2-like 1] | 2.75 | 2.76E-02 |
| 1990 | CELA1 [chymotrypsin-like elastase family, member 1] | 2.75 | 2.53E-02 |
| 51226 | COPZ2 [coatomer protein complex, subunit zeta 2] | 2.74 | 4.52E-03 |
| 51412 | ACTL6B [actin-like 6B] | 2.74 | 4.28E-03 |
| 6372 | CXCL6 [chemokine (C-X-C motif) ligand 6] | 2.74 | 2.71E-02 |
| 23462 | HEY1 [hairy/enhancer-of-split related with YRPW motif 1] | 2.74 | 8.93E-03 |
| 3557 | IL1RN [interleukin 1 receptor antagonist] | 2.71 | 1.70E-04 |
| 797 | CALCB [calcitonin-related polypeptide beta] | 2.65 | 1.11E-02 |
| 92691 | TMEM169 [transmembrane protein 169] | 2.65 | 8.15E-03 |
| 134147 | CMBL [carboxymethylenebutenolidase homolog] | 2.63 | 2.65E-02 |
| 161253 | REM2 [RAS (RAD and GEM)-like GTP binding 2] | 2.62 | 9.46E-03 |
| 115362 | GBP5 [guanylate binding protein 5] | 2.62 | 2.58E-04 |
| 147872 | CCDC155 [coiled-coil domain containing 155] | 2.61 | 2.10E-02 |
| 9001 | HAP1 [huntingtin-associated protein 1] | 2.61 | 1.34E-02 |
| 8277 | TKTL1 [transketolase-like 1] | 2.61 | 5.18E-04 |
| 6348 | CCL3 [chemokine (C-C motif) ligand 3] | 2.60 | 2.92E-04 |
| 402665 | IGLON5 [IgLON family member 5] | 2.60 | 4.38E-02 |
| 283208 | P4HA3 [prolyl 4-hydroxylase, alpha polypeptide III] | 2.60 | 4.81E-02 |
| 55679 | LIMS2 [LIM and senescent cell antigen-like domains 2] | 2.59 | 2.05E-02 |
| 55740 | ENAH [enabled homolog (Drosophila)] | 2.59 | 4.09E-03 |
| 1493 | CTLA4 [cytotoxic T-lymphocyte-associated protein 4] | 2.58 | 3.68E-02 |
| 51725 | FBXO40 [F-box protein 40] | 2.58 | 9.67E-03 |
| 3783 | KCNN4 [potassium intermediate/small conductance channel] | 2.57 | 3.99E-04 |
| 441326 | FAM90A18 [family with sequence similarity 90, member A18] | 2.57 | 1.59E-02 |
| 441328 | FAM90A10 [family with sequence similarity 90, member A10] | 2.57 | 1.59E-02 |
| 728753 | FAM90A19 [family with sequence similarity 90, member A19] | 2.57 | 1.59E-02 |
| 6355 | CCL8 [chemokine (C-C motif) ligand 8] | 2.56 | 4.38E-03 |
| 100287898 | TTC34 [tetratricopeptide repeat domain 34] | 2.56 | 1.30E-02 |
| 6004 | RGS16 [regulator of G-protein signaling 16] | 2.56 | 4.99E-02 |
| 140032 | RPS4Y2 [ribosomal protein S4, Y-linked 2] | 2.52 | 1.27E-02 |
| 10815 | CPLX1 [complexin 1] | 2.51 | 5.62E-03 |
| 85411 | C6orf114 [chromosome 6 open reading frame 114] | 2.51 | 3.45E-02 |
| 22822 | PHLDA1 [pleckstrin homology-like domain, family A, 1] | 2.51 | 4.70E-04 |
| 60506 | NYX [nyctalopin] | 2.50 | 8.44E-03 |
| 161829 | EXD1 [exonuclease 3'-5' domain containing 1] | 2.50 | 4.97E-03 |
| 92162 | TMEM88 [transmembrane protein 88] | 2.50 | 1.79E-03 |
| 83715 | ESPN [espin] | 2.50 | 9.57E-03 |
| 114770 | PGLYRP2 [peptidoglycan recognition protein 2] | 2.49 | 4.74E-02 |
| 1728 | NQO1 [NAD(P)H dehydrogenase, quinone 1] | 2.49 | 1.12E-02 |
| 154043 | CNKSR3 [CNKSR family member 3] | 2.49 | 3.03E-02 |
| 10485 | C1orf61 [chromosome 1 open reading frame 61] | 2.49 | 2.92E-03 |
| 140807 | KRT72 [keratin 72] | 2.48 | 1.34E-03 |
| 2920 | CXCL2 [chemokine (C-X-C motif) ligand 2] | 2.47 | 6.40E-04 |
| 10046 | MAMLD1 [mastermind-like domain containing 1] | 2.46 | 3.00E-03 |
| 146177 | VWA3A [von Willebrand factor A domain containing 3A] | 2.46 | 1.12E-02 |
| 6003 | RGS13 [regulator of G-protein signaling 13] | 2.46 | 4.99E-02 |
| 1071 | CETP [cholesteryl ester transfer protein, plasma] | 2.46 | 7.67E-03 |
| 6347 | CCL2 [chemokine (C-C motif) ligand 2] | 2.46 | 7.59E-04 |
| 91147 | TMEM67 [transmembrane protein 67] | 2.45 | 7.45E-03 |
| 5146 | PDE6C [phosphodiesterase 6C, cGMP-specific, cone, α prime] | 2.45 | 3.12E-02 |
| 658 | BMPR1B [bone morphogenetic protein receptor, type IB] | 2.45 | 3.23E-02 |
| 55966 | AJAP1 [adherens junctions associated protein 1] | 2.45 | 4.59E-02 |
| 3798 | KIF5A [kinesin family member 5A] | 2.45 | 4.59E-02 |
| 55848 | C9orf46 [chromosome 9 open reading frame 46] | 2.44 | 2.10E-03 |
| 117532 | TMC2 [transmembrane channel-like 2] | 2.44 | 1.76E-02 |
| 5913 | RAPSN [receptor-associated protein of the synapse] | 2.43 | 5.90E-03 |
| 3569 | IL6 [interleukin 6 (interferon, beta 2)] | 2.42 | 3.44E-02 |
| 64109 | CRLF2 [cytokine receptor-like factor 2] | 2.42 | 1.62E-03 |
| 84261 | FBXW9 [F-box and WD repeat domain containing 9] | 2.42 | 7.81E-03 |
| 1289 | COL5A1 [collagen, type V, alpha 1] | 2.40 | 2.13E-02 |
| 9133 | CCNB2 [cyclin B2] | 2.40 | 1.96E-02 |
| 117247 | SLC16A10 [solute carrier family 16, member 10] | 2.39 | 2.01E-03 |
| 283638 | KIAA0284 [KIAA0284] | 2.38 | 3.12E-03 |
| 221416 | C6orf223 [chromosome 6 open reading frame 223] | 2.38 | 1.48E-03 |
| 80343 | SEL1L2 [sel-1 suppressor of lin-12-like 2 (C. elegans)] | 2.37 | 3.85E-02 |
| 255352 | C10orf93 [chromosome 10 open reading frame 93] | 2.37 | 8.66E-03 |
| 54777 | C10orf92 [chromosome 10 open reading frame 92] | 2.37 | 8.66E-03 |
| 80326 | WNT10A [wingless-type MMTV integration site family, 10A] | 2.37 | 4.22E-03 |
| 414236 | C10orf55 [chromosome 10 open reading frame 55] | 2.36 | 3.72E-03 |
| 3090 | HIC1 [hypermethylated in cancer 1] | 2.36 | 1.93E-03 |
| 161502 | C15orf26 [chromosome 15 open reading frame 26] | 2.35 | 1.72E-02 |
| 7124 | TNF [tumor necrosis factor] | 2.35 | 1.46E-03 |
| 8828 | NRP2 [neuropilin 2] | 2.34 | 2.40E-03 |
| 10630 | PDPN [podoplanin] | 2.33 | 1.22E-02 |
| 25822 | DNAJB5 [DnaJ (Hsp40) homolog, subfamily B, member 5] | 2.32 | 1.83E-03 |
| 4318 | MMP9 [matrix metallopeptidase 9] | 2.31 | 1.42E-03 |
| 5292 | PIM1 [pim-1 oncogene] | 2.31 | 1.63E-03 |
| 51330 | TNFRSF12A [tumor necrosis factor receptor superfamily, 12A] | 2.30 | 3.31E-03 |
| 114822 | RHPN1 [rhophilin, Rho GTPase binding protein 1] | 2.30 | 3.36E-03 |
| 597 | BCL2A1 [BCL2-related protein A1] | 2.29 | 1.68E-03 |
| 65268 | WNK2 [WNK lysine deficient protein kinase 2] | 2.29 | 1.74E-02 |
| 284119 | PTRF [polymerase I and transcript release factor] | 2.29 | 2.68E-03 |
| 9856 | KIAA0319 [KIAA0319] | 2.28 | 2.19E-02 |
| 401097 | LOC401097 [Similar to LOC166075] | 2.28 | 4.74E-02 |
| 4234 | METTL1 [methyltransferase like 1] | 2.27 | 1.09E-02 |
| 11001 | SLC27A2 [solute carrier family 27, member 2] | 2.27 | 1.54E-02 |
| 4137 | MAPT [microtubule-associated protein tau] | 2.26 | 3.67E-02 |
| 6772 | STAT1 [signal transducer and activator of transcription 1] | 2.26 | 1.91E-03 |
| 57449 | PLEKHG5 [pleckstrin domain containing, family G, 5] | 2.25 | 4.74E-02 |
| 1906 | EDN1 [endothelin 1] | 2.25 | 1.20E-02 |
| 401409 | RAB19 [RAB19, member RAS oncogene family] | 2.24 | 3.54E-02 |
| 5557 | PRIM1 [primase, DNA, polypeptide 1 (49kDa)] | 2.24 | 1.04E-02 |
| 23495 | TNFRSF13B [tumor necrosis factor receptor superfamily, 13B] | 2.24 | 1.30E-02 |
| 728116 | ZBTB8B [zinc finger and BTB domain containing 8B] | 2.23 | 2.66E-02 |
| 154664 | ABCA13 [ATP-binding cassette, sub-family A (ABC1), 13] | 2.23 | 2.43E-03 |
| 135228 | CD109 [CD109 molecule] | 2.23 | 2.53E-03 |
| 57628 | DPP10 [dipeptidyl-peptidase 10 (non-functional)] | 2.23 | 2.26E-03 |
| 58157 | NGB [neuroglobin] | 2.23 | 4.16E-02 |
| 135 | ADORA2A [adenosine A2a receptor] | 2.23 | 2.50E-03 |
| 400746 | C1orf130 [chromosome 1 open reading frame 130] | 2.22 | 2.15E-02 |
| 9891 | NUAK1 [NUAK family, SNF1-like kinase, 1] | 2.22 | 2.10E-02 |
| 129807 | NEU4 [sialidase 4] | 2.22 | 3.71E-03 |
| 1768 | DNAH6 [dynein, axonemal, heavy chain 6] | 2.21 | 2.55E-03 |
| 388372 | CCL4L2 [chemokine (C-C motif) ligand 4-like 2] | 2.21 | 2.63E-03 |
| 9560 | CCL4L1 [chemokine (C-C motif) ligand 4-like 1] | 2.21 | 2.63E-03 |
| 3939 | LDHA [lactate dehydrogenase A] | 2.21 | 2.44E-03 |
| 116211 | TM4SF19 [transmembrane 4 L six family member 19] | 2.21 | 4.73E-03 |
| 6369 | CCL24 [chemokine (C-C motif) ligand 24] | 2.21 | 2.83E-03 |
| 414062 | CCL3L3 [chemokine (C-C motif) ligand 3-like 3] | 2.21 | 2.49E-03 |
| 6349 | CCL3L1 [chemokine (C-C motif) ligand 3-like 1] | 2.21 | 2.49E-03 |
| 10993 | SDS [serine dehydratase] | 2.20 | 4.12E-03 |
| 666 | BOK [BCL2-related ovarian killer] | 2.19 | 2.79E-02 |
| 26330 | GAPDHS [glyceraldehyde-3-phosphate dehydrogenase, spermatogenic] | 2.19 | 1.72E-02 |
| 1514 | CTSL1 [cathepsin L1] | 2.18 | 2.88E-03 |
| 784 | CACNB3 [calcium channel, voltage-dependent, beta 3 subunit] | 2.18 | 3.21E-02 |
| 8651 | SOCS1 [suppressor of cytokine signaling 1] | 2.17 | 7.25E-03 |
| 5133 | PDCD1 [programmed cell death 1] | 2.16 | 4.30E-03 |
| 3208 | HPCA [hippocalcin] | 2.16 | 2.72E-02 |
| 200958 | MUC20 [mucin 20, cell surface associated] | 2.16 | 2.73E-02 |
| 80270 | HSD3B7 [hydroxy-δ-5-steroid dehydrog, 3 β- and steroid δ-isomerase 7] | 2.15 | 9.17E-03 |
| 3142 | HLX [H2.0-like homeobox] | 2.15 | 3.64E-03 |
| 23764 | MAFF [v-maf musculoaponeurotic fibrosarcoma oncogene homolog F] | 2.14 | 3.76E-03 |
| 146862 | UNC45B [unc-45 homolog B (C. elegans)] | 2.12 | 3.93E-03 |
| 5311 | PKD2 [polycystic kidney disease 2 (autosomal dominant)] | 2.12 | 4.92E-03 |
| 116444 | GRIN3B [glutamate receptor, ionotropic, N-methyl-D-aspartate 3B] | 2.11 | 1.37E-02 |
| 916 | CD3E [CD3e molecule, epsilon (CD3-TCR complex)] | 2.11 | 1.12E-02 |
| 2635 | GBP3 [guanylate binding protein 3] | 2.10 | 5.11E-03 |
| 10516 | FBLN5 [fibulin 5] | 2.10 | 3.01E-02 |
| 4356 | MPP3 [membrane protein, palmitoylated 3 (MAGUK p55 subfamily # 3)] | 2.10 | 4.49E-02 |
| 3750 | KCND1 [potassium voltage-gated channel, Shal-related subfam., member 1] | 2.09 | 1.87E-02 |
| 9942 | XYLB [xylulokinase homolog (H. influenzae)] | 2.09 | 4.60E-02 |
| 6351 | CCL4 [chemokine (C-C motif) ligand 4] | 2.08 | 5.08E-03 |
| 100130311 | C17orf107 [chromosome 17 open reading frame 107] | 2.08 | 6.95E-03 |
| 5328 | PLAU [plasminogen activator, urokinase] | 2.08 | 1.63E-02 |
| 81615 | TMEM163 [transmembrane protein 163] | 2.08 | 2.73E-02 |
| 201305 | SPNS3 [spinster homolog 3 (Drosophila)] | 2.08 | 9.76E-03 |
| 55721 | IQCC [IQ motif containing C] | 2.07 | 3.41E-02 |
| 400509 | RUNDC2B [RUN domain containing 2B] | 2.06 | 1.21E-02 |
| 7071 | KLF10 [Kruppel-like factor 10] | 2.05 | 6.45E-03 |
| 80830 | APOL6 [apolipoprotein L, 6] | 2.05 | 6.69E-03 |
| 2070 | EYA4 [eyes absent homolog 4 (Drosophila)] | 2.04 | 6.50E-03 |
| 4616 | GADD45B [growth arrest and DNA-damage-inducible, beta] | 2.03 | 7.34E-03 |
| 7035 | TFPI [tissue factor pathway inhibitor] | 2.03 | 3.63E-02 |
| 89886 | SLAMF9 [SLAM family member 9] | 2.01 | 1.49E-02 |
| 93474 | ZNF670 [zinc finger protein 670] | 2.01 | 2.93E-02 |
| 50512 | PODXL2 [podocalyxin-like 2] | 2.01 | 4.59E-02 |
| 10579 | TACC2 [transforming, acidic coiled-coil containing protein 2] | 2.01 | 3.34E-02 |
| 3640 | INSL3 [insulin-like 3 (Leydig cell)] | 2.00 | 4.64E-02 |
| 28984 | C13orf15 [chromosome 13 open reading frame 15] | 2.00 | 8.38E-03 |
| 1839 | HBEGF [heparin-binding EGF-like growth factor] | 2.00 | 8.16E-03 |
| 4923 | NTSR1 [neurotensin receptor 1 (high affinity)] | 2.00 | 8.90E-03 |
| 8871 | SYNJ2 [synaptojanin 2] | 2.00 | 1.05E-02 |
| 9452 | ITM2A [integral membrane protein 2A] | 1.99 | 2.16E-02 |
| 319101 | KRT73 [keratin 73] | 1.99 | 1.95E-02 |
| 9953 | HS3ST3B1 [heparan sulfate (glucosamine) 3-O-sulfotransferase 3B1] | 1.99 | 1.32E-02 |
| 84166 | NLRC5 [NLR family, CARD domain containing 5] | 1.99 | 8.53E-03 |
| 2867 | FFAR2 [free fatty acid receptor 2] | 1.99 | 9.69E-03 |
| 8913 | CACNA1G [calcium channel, voltage-dependent, T type, alpha 1G subunit] | 1.99 | 2.01E-02 |
| 1740 | DLG2 [discs, large homolog 2 (Drosophila)] | 1.99 | 8.42E-03 |
| 6354 | CCL7 [chemokine (C-C motif) ligand 7] | 1.99 | 1.93E-02 |
| 54903 | MKS1 [Meckel syndrome, type 1] | 1.98 | 2.22E-02 |
| 5470 | PPEF2 [protein phosphatase, EF-hand calcium binding domain 2] | 1.97 | 9.51E-03 |
| 23413 | NCS1 [neuronal calcium sensor 1] | 1.97 | 1.80E-02 |
| 388646 | GBP7 [guanylate binding protein 7] | 1.97 | 2.59E-02 |
| 4826 | NNAT [neuronatin] | 1.97 | 3.04E-02 |
| 83714 | NRIP2 [nuclear receptor interacting protein 2] | 1.97 | 2.46E-02 |
| 642103 | LOC642103 [similar to Maltase-glucoamylase, intestinal] | 1.96 | 9.66E-03 |
| 8972 | MGAM [maltase-glucoamylase (alpha-glucosidase)] | 1.96 | 9.66E-03 |
| 7298 | TYMS [thymidylate synthetase] | 1.96 | 2.98E-02 |
| 1776 | DNASE1L3 [deoxyribonuclease I-like 3] | 1.95 | 2.34E-02 |
| 282966 | C10orf53 [chromosome 10 open reading frame 53] | 1.94 | 1.51E-02 |
| 114904 | C1QTNF6 [C1q and tumor necrosis factor related protein 6] | 1.94 | 3.44E-02 |
| 57575 | PCDH10 [protocadherin 10] | 1.94 | 1.69E-02 |
| 85300 | ATCAY [ataxia, cerebellar, Cayman type] | 1.94 | 3.30E-02 |
| 414060 | TBC1D3C [TBC1 domain family, member 3C] | 1.94 | 1.12E-02 |
| 729877 | TBC1D3H [TBC1 domain family, member 3H] | 1.94 | 1.12E-02 |
| 81796 | SLCO5A1 [solute carrier organic anion transporter family, member 5A1] | 1.93 | 1.14E-02 |
| 6804 | STX1A [syntaxin 1A (brain)] | 1.92 | 2.20E-02 |
| 3809 | KIR2DS4 [killer cell Ig-like receptor, two domains, short cytopl. tail, 4] | 1.92 | 2.12E-02 |
| 3576 | IL8 [interleukin 8] | 1.92 | 1.21E-02 |
| 57541 | ZNF398 [zinc finger protein 398] | 1.92 | 1.52E-02 |
| 575 | BAI1 [brain-specific angiogenesis inhibitor 1] | 1.92 | 1.44E-02 |
| 3595 | IL12RB2 [interleukin 12 receptor, beta 2] | 1.91 | 2.11E-02 |
| 23601 | CLEC5A [C-type lectin domain family 5, member A] | 1.91 | 1.29E-02 |
| 149478 | BTBD19 [BTB (POZ) domain containing 19] | 1.90 | 1.61E-02 |
| 10936 | GPR75 [G protein-coupled receptor 75] | 1.90 | 3.86E-02 |
| 9021 | SOCS3 [suppressor of cytokine signaling 3] | 1.90 | 1.42E-02 |
| 414308 | MRC1L1 [mannose receptor, C type 1-like 1] | 1.90 | 2.98E-02 |
| 23483 | TGDS [TDP-glucose 4,6-dehydratase] | 1.90 | 3.88E-02 |
| 5603 | MAPK13 [mitogen-activated protein kinase 13] | 1.90 | 1.87E-02 |
| 84444 | DOT1L [DOT1-like, histone H3 methyltransferase] | 1.89 | 1.48E-02 |
| 5222 | PGA5 [pepsinogen 5, group I (pepsinogen A)] | 1.88 | 3.48E-02 |
| 643847 | PGA4 [pepsinogen 4, group I (pepsinogen A)] | 1.88 | 3.48E-02 |
| 649034 | LOC649034 [similar to Pepsin A precursor] | 1.88 | 3.48E-02 |
| 170371 | C10orf128 [chromosome 10 open reading frame 128] | 1.88 | 1.84E-02 |
| 161882 | ZFPM1 [zinc finger protein, multitype 1] | 1.88 | 1.94E-02 |
| 2317 | FLNB [filamin B, beta] | 1.88 | 1.54E-02 |
| 6676 | SPAG4 [sperm associated antigen 4] | 1.88 | 4.39E-02 |
| 115653 | KIR3DL3 [killer cell Ig-like receptor, 3 domains, long cytoplasmic tail, 3] | 1.88 | 3.53E-02 |
| 113730 | KLHDC7B [kelch domain containing 7B] | 1.87 | 4.02E-02 |
| 54206 | ERRFI1 [ERBB receptor feedback inhibitor 1] | 1.87 | 2.34E-02 |
| 54210 | TREM1 [triggering receptor expressed on myeloid cells 1] | 1.87 | 1.64E-02 |
| 83999 | KREMEN1 [kringle containing transmembrane protein 1] | 1.87 | 2.16E-02 |
| 3811 | KIR3DL1 [killer cell Ig-like receptor, three domains, long cytopl. tail, 1] | 1.86 | 2.06E-02 |
| 3812 | KIR3DL2 [killer cell Ig-like receptor, three domains, long cytopl. tail, 2] | 1.86 | 2.06E-02 |
| 100132285 | KIR2DS2 [killer cell Ig-like receptor, two domains, short cytopl. tail, 2] | 1.86 | 2.07E-02 |
| 3804 | KIR2DL3 [killer cell Ig-like receptor, two domains, long cytoplasmic tail, 3] | 1.86 | 2.07E-02 |
| 4983 | OPHN1 [oligophrenin 1] | 1.85 | 1.78E-02 |
| 9985 | REC8 [REC8 homolog (yeast)] | 1.85 | 2.27E-02 |
| 5214 | PFKP [phosphofructokinase, platelet] | 1.85 | 1.87E-02 |
| 8829 | NRP1 [neuropilin 1] | 1.84 | 2.13E-02 |
| 165530 | CLEC4F [C-type lectin domain family 4, member F] | 1.84 | 3.58E-02 |
| 4360 | MRC1 [mannose receptor, C type 1] | 1.84 | 3.93E-02 |
| 27074 | LAMP3 [lysosomal-associated membrane protein 3] | 1.84 | 4.14E-02 |
| 23780 | APOL2 [apolipoprotein L, 2] | 1.84 | 2.03E-02 |
| 64210 | MMS19 [MMS19 nucleotide excision repair homolog (S. cerevisiae)] | 1.84 | 2.10E-02 |
| 445347 | TARP [TCR gamma alternate reading frame protein] | 1.84 | 2.78E-02 |
| 8061 | FOSL1 [FOS-like antigen 1] | 1.83 | 3.59E-02 |
| 26025 | PCDHGA12 [protocadherin gamma subfamily A, 12] | 1.83 | 4.23E-02 |
| 5098 | PCDHGC3 [protocadherin gamma subfamily C, 3] | 1.83 | 4.23E-02 |
| 56097 | PCDHGC5 [protocadherin gamma subfamily C, 5] | 1.83 | 4.23E-02 |
| 56098 | PCDHGC4 [protocadherin gamma subfamily C, 4] | 1.83 | 4.23E-02 |
| 56099 | PCDHGB7 [protocadherin gamma subfamily B, 7] | 1.83 | 4.23E-02 |
| 56100 | PCDHGB6 [protocadherin gamma subfamily B, 6] | 1.83 | 4.23E-02 |
| 56101 | PCDHGB5 [protocadherin gamma subfamily B, 5] | 1.83 | 4.23E-02 |
| 56102 | PCDHGB3 [protocadherin gamma subfamily B, 3] | 1.83 | 4.23E-02 |
| 56103 | PCDHGB2 [protocadherin gamma subfamily B, 2] | 1.83 | 4.23E-02 |
| 56104 | PCDHGB1 [protocadherin gamma subfamily B, 1] | 1.83 | 4.23E-02 |
| 56105 | PCDHGA11 [protocadherin gamma subfamily A, 11] | 1.83 | 4.23E-02 |
| 56106 | PCDHGA10 [protocadherin gamma subfamily A, 10] | 1.83 | 4.23E-02 |
| 56107 | PCDHGA9 [protocadherin gamma subfamily A, 9] | 1.83 | 4.23E-02 |
| 56108 | PCDHGA7 [protocadherin gamma subfamily A, 7] | 1.83 | 4.23E-02 |
| 56109 | PCDHGA6 [protocadherin gamma subfamily A, 6] | 1.83 | 4.23E-02 |
| 56110 | PCDHGA5 [protocadherin gamma subfamily A, 5] | 1.83 | 4.23E-02 |
| 56111 | PCDHGA4 [protocadherin gamma subfamily A, 4] | 1.83 | 4.23E-02 |
| 56112 | PCDHGA3 [protocadherin gamma subfamily A, 3] | 1.83 | 4.23E-02 |
| 56113 | PCDHGA2 [protocadherin gamma subfamily A, 2] | 1.83 | 4.23E-02 |
| 56114 | PCDHGA1 [protocadherin gamma subfamily A, 1] | 1.83 | 4.23E-02 |
| 8641 | PCDHGB4 [protocadherin gamma subfamily B, 4] | 1.83 | 4.23E-02 |
| 9708 | PCDHGA8 [protocadherin gamma subfamily A, 8] | 1.83 | 4.23E-02 |
| 165186 | FAM179A [family with sequence similarity 179, member A] | 1.83 | 2.87E-02 |
| 80176 | SPSB1 [splA/ryanodine receptor domain and SOCS box containing 1] | 1.82 | 3.57E-02 |
| 6319 | SCD [stearoyl-CoA desaturase (delta-9-desaturase)] | 1.82 | 2.57E-02 |
| 8784 | TNFRSF18 [tumor necrosis factor receptor superfamily, member 18] | 1.82 | 3.18E-02 |
| 51056 | LAP3 [leucine aminopeptidase 3] | 1.81 | 2.29E-02 |
| 92610 | TIFA [TRAF-interacting protein with forkhead-associated domain] | 1.81 | 3.29E-02 |
| 64061 | TSPYL2 [TSPY-like 2] | 1.81 | 2.32E-02 |
| 770 | CA11 [carbonic anhydrase XI] | 1.81 | 4.65E-02 |
| 6262 | RYR2 [ryanodine receptor 2 (cardiac)] | 1.81 | 2.28E-02 |
| 84069 | PLEKHN1 [pleckstrin homology domain containing, family N member 1] | 1.81 | 4.99E-02 |
| 64386 | MMP25 [matrix metallopeptidase 25] | 1.81 | 2.42E-02 |
| 4665 | NAB2 [NGFI-A binding protein 2 (EGR1 binding protein 2)] | 1.80 | 2.40E-02 |
| 79836 | LONRF3 [LON peptidase N-terminal domain and ring finger 3] | 1.80 | 2.92E-02 |
| 1145 | CHRNE [cholinergic receptor, nicotinic, epsilon] | 1.80 | 2.64E-02 |
| 6550 | SLC9A3 [solute carrier family 9 (sodium/hydrogen exchanger), member 3] | 1.80 | 2.55E-02 |
| 9047 | SH2D2A [SH2 domain containing 2A] | 1.80 | 2.61E-02 |
| 53831 | GPR84 [G protein-coupled receptor 84] | 1.80 | 2.73E-02 |
| 9308 | CD83 [CD83 molecule] | 1.79 | 2.49E-02 |
| 124637 | CYB5D1 [cytochrome b5 domain containing 1] | 1.79 | 3.62E-02 |
| 4023 | LPL [lipoprotein lipase] | 1.79 | 3.51E-02 |
| 6383 | SDC2 [syndecan 2] | 1.79 | 2.58E-02 |
| 10538 | BATF [basic leucine zipper transcription factor, ATF-like] | 1.79 | 4.23E-02 |
| 10011 | SRA1 [steroid receptor RNA activator 1] | 1.79 | 2.60E-02 |
| 92105 | INTS4 [integrator complex subunit 4] | 1.78 | 2.62E-02 |
| 2171 | FABP5 [fatty acid binding protein 5 (psoriasis-associated)] | 1.78 | 2.83E-02 |
| 140699 | C20orf132 [chromosome 20 open reading frame 132] | 1.78 | 3.45E-02 |
| 3164 | NR4A1 [nuclear receptor subfamily 4, group A, member 1] | 1.77 | 2.83E-02 |
| 22914 | KLRK1 [killer cell lectin-like receptor subfamily K, member 1] | 1.77 | 3.00E-02 |
| 140701 | C20orf135 [chromosome 20 open reading frame 135] | 1.77 | 3.45E-02 |
| 9672 | SDC3 [syndecan 3] | 1.77 | 4.19E-02 |
| 8653 | DDX3Y [DEAD (Asp-Glu-Ala-Asp) box polypeptide 3, Y-linked] | 1.77 | 2.87E-02 |
| 644815 | FAM83G [family with sequence similarity 83, member G] | 1.76 | 4.20E-02 |
| 1263 | PLK3 [polo-like kinase 3] | 1.76 | 3.14E-02 |
| 3914 | LAMB3 [laminin, beta 3] | 1.76 | 3.23E-02 |
| 6768 | ST14 [suppression of tumorigenicity 14 (colon carcinoma)] | 1.75 | 3.34E-02 |
| 83666 | PARP9 [poly (ADP-ribose) polymerase family, member 9] | 1.75 | 3.35E-02 |
| 114132 | SIGLEC11 [sialic acid binding Ig-like lectin 11] | 1.74 | 3.20E-02 |
| 1130 | LYST [lysosomal trafficking regulator] | 1.74 | 3.25E-02 |
| 10938 | EHD1 [EH-domain containing 1] | 1.74 | 3.29E-02 |
| 2672 | GFI1 [growth factor independent 1 transcription repressor] | 1.74 | 4.49E-02 |
| 8460 | TPST1 [tyrosylprotein sulfotransferase 1] | 1.74 | 4.18E-02 |
| 200734 | SPRED2 [sprouty-related, EVH1 domain containing 2] | 1.74 | 3.89E-02 |
| 8767 | RIPK2 [receptor-interacting serine-threonine kinase 2] | 1.74 | 3.81E-02 |
| 604 | BCL6 [B-cell CLL/lymphoma 6] | 1.73 | 3.43E-02 |
| 23101 | MCF2L2 [MCF.2 cell line derived transforming sequence-like 2] | 1.73 | 3.69E-02 |
| 285966 | FAM115C [family with sequence similarity 115, member C] | 1.73 | 3.55E-02 |
| 7052 | TGM2 [transglutaminase 2, protein-glutamine-gamma-glutamyltransferase)] | 1.73 | 3.72E-02 |
| 2444 | FRK [fyn-related kinase] | 1.73 | 3.66E-02 |
| 51332 | SPTBN5 [spectrin, beta, non-erythrocytic 5] | 1.72 | 4.24E-02 |
| 6890 | TAP1 [transporter 1, ATP-binding cassette, sub-family B (MDR/TAP)] | 1.72 | 3.63E-02 |
| 56978 | PRDM8 [PR domain containing 8] | 1.72 | 4.54E-02 |
| 164045 | HFM1 [HFM1, ATP-dependent DNA helicase homolog (S. cerevisiae)] | 1.72 | 3.64E-02 |
| 24145 | PANX1 [pannexin 1] | 1.72 | 4.50E-02 |
| 80183 | C13orf18 [chromosome 13 open reading frame 18] | 1.72 | 3.92E-02 |
| 83858 | ATAD3B [ATPase family, AAA domain containing 3B] | 1.72 | 4.04E-02 |
| 728841 | NBPF8 [neuroblastoma breakpoint family, member 8] | 1.72 | 3.73E-02 |
| 8398 | PLA2G6 [phospholipase A2, group VI (cytosolic, calcium-independent)] | 1.71 | 4.36E-02 |
| 10221 | TRIB1 [tribbles homolog 1 (Drosophila)] | 1.71 | 3.88E-02 |
| 2208 | FCER2 [Fc fragment of IgE, low affinity II, receptor for (CD23)] | 1.71 | 4.56E-02 |
| 9235 | IL32 [interleukin 32] | 1.71 | 4.52E-02 |
| 79703 | C11orf80 [chromosome 11 open reading frame 80] | 1.71 | 4.88E-02 |
| 3805 | KIR2DL4 [killer cell Ig-like receptor, two domains, long cytoplasmic tail, 4] | 1.71 | 4.24E-02 |
| 1958 | EGR1 [early growth response 1] | 1.70 | 4.01E-02 |
| 540 | ATP7B [ATPase, Cu++ transporting, beta polypeptide] | 1.70 | 4.39E-02 |
| 728294 | D2HGDH [D-2-hydroxyglutarate dehydrogenase] | 1.70 | 4.39E-02 |
| 26086 | GPSM1 [G-protein signaling modulator 1] | 1.69 | 4.80E-02 |
| 3681 | ITGAD [integrin, alpha D] | 1.69 | 4.46E-02 |
| 91351 | DDX60L [DEAD (Asp-Glu-Ala-Asp) box polypeptide 60-like] | 1.69 | 4.49E-02 |
| 100131635 | LOC100131635 [hCG1645011-like] | 1.69 | 4.48E-02 |
| 747 | DAGLA [diacylglycerol lipase, alpha] | 1.69 | 4.76E-02 |
| 22809 | ATF5 [activating transcription factor 5] | 1.68 | 4.45E-02 |
| 84807 | NFKBID [nuclear factor κ polypeptide gene enhancer in B-cells inhibitor, δ] | 1.68 | 4.69E-02 |
| 9172 | MYOM2 [myomesin (M-protein) 2, 165kDa] | 1.68 | 4.98E-02 |
| 5468 | PPARG [peroxisome proliferator-activated receptor gamma] | 1.68 | 4.77E-02 |
| 10379 | IRF9 [interferon regulatory factor 9] | 1.68 | 4.75E-02 |
| 117289 | TAGAP [T-cell activation RhoGTPase activating protein] | 1.68 | 4.86E-02 |
| 8877 | SPHK1 [sphingosine kinase 1] | 1.67 | 5.00E-02 |
| 6515 | SLC2A3 [solute carrier family 2 (facilitated glucose transporter), member 3] | 1.67 | 4.84E-02 |
| 11237 | RNF24 [ring finger protein 24] | -1.68 | 4.88E-02 |
| 10129 | FRY [furry homolog (Drosophila)] | -1.69 | 4.49E-02 |
| 23355 | VPS8 [vacuolar protein sorting 8 homolog (S. cerevisiae)] | -1.69 | 4.83E-02 |
| 202309 | GAPT [GRB2-binding adaptor protein, transmembrane] | -1.70 | 4.21E-02 |
| 64231 | MS4A6A [membrane-spanning 4-domains, subfamily A, member 6A] | -1.70 | 4.57E-02 |
| 5166 | PDK4 [pyruvate dehydrogenase kinase, isozyme 4] | -1.71 | 4.88E-02 |
| 84542 | KIAA1841 [KIAA1841] | -1.71 | 4.95E-02 |
| 1955 | MEGF9 [multiple EGF-like-domains 9] | -1.72 | 3.85E-02 |
| 4778 | NFE2 [nuclear factor (erythroid-derived 2), 45kDa] | -1.72 | 4.04E-02 |
| 51099 | ABHD5 [abhydrolase domain containing 5] | -1.73 | 4.11E-02 |
| 6252 | RTN1 [reticulon 1] | -1.73 | 3.60E-02 |
| 9586 | CREB5 [cAMP responsive element binding protein 5] | -1.74 | 4.47E-02 |
| 81537 | SGPP1 [sphingosine-1-phosphate phosphatase 1] | -1.75 | 4.26E-02 |
| 401494 | PTPLAD2 [protein tyrosine phosphatase-like A domain containing 2] | -1.76 | 3.98E-02 |
| 11167 | FSTL1 [follistatin-like 1] | -1.76 | 4.93E-02 |
| 8876 | VNN1 [vanin 1] | -1.76 | 3.30E-02 |
| 2651 | GCNT2 [glucosaminyl (N-acetyl) transferase 2, I-branching enzyme] | -1.76 | 4.66E-02 |
| 3613 | IMPA2 [inositol(myo)-1(or 4)-monophosphatase 2] | -1.77 | 3.95E-02 |
| 115106 | HAUS1 [HAUS augmin-like complex, subunit 1] | -1.77 | 4.91E-02 |
| 64115 | C10orf54 [chromosome 10 open reading frame 54] | -1.77 | 2.80E-02 |
| 7644 | ZNF91 [zinc finger protein 91] | -1.78 | 3.68E-02 |
| 201799 | TMEM154 [transmembrane protein 154] | -1.78 | 2.97E-02 |
| 55 | ACPP [acid phosphatase, prostate] | -1.79 | 4.27E-02 |
| 9936 | CD302 [CD302 molecule] | -1.79 | 2.72E-02 |
| 11200 | CHEK2 [CHK2 checkpoint homolog (S. pombe)] | -1.80 | 4.89E-02 |
| 55610 | CCDC132 [coiled-coil domain containing 132] | -1.80 | 3.62E-02 |
| 51313 | FAM198B [family with sequence similarity 198, member B] | -1.80 | 2.41E-02 |
| 54675 | CRLS1 [cardiolipin synthase 1] | -1.80 | 3.17E-02 |
| 84446 | BRSK1 [BR serine/threonine kinase 1] | -1.80 | 4.99E-02 |
| 8526 | DGKE [diacylglycerol kinase, epsilon 64kDa] | -1.81 | 3.85E-02 |
| 6403 | SELP [selectin P (granule membrane protein 140kDa, antigen CD62)] | -1.82 | 4.25E-02 |
| 25976 | TIPARP [TCDD-inducible poly(ADP-ribose) polymerase] | -1.83 | 2.16E-02 |
| 8342 | HIST1H2BM [histone cluster 1, H2bm] | -1.83 | 4.65E-02 |
| 5738 | PTGFRN [prostaglandin F2 receptor negative regulator] | -1.83 | 2.10E-02 |
| 23171 | GPD1L [glycerol-3-phosphate dehydrogenase 1-like] | -1.84 | 4.01E-02 |
| 5783 | PTPN13 [protein tyrosine phosphatase, non-receptor type 13] | -1.85 | 4.85E-02 |
| 164091 | PAQR7 [progestin and adipoQ receptor family member VII] | -1.85 | 2.45E-02 |
| 8464 | SUPT3H [suppressor of Ty 3 homolog (S. cerevisiae)] | -1.85 | 4.95E-02 |
| 7122 | CLDN5 [claudin 5] | -1.86 | 3.04E-02 |
| 3274 | HRH2 [histamine receptor H2] | -1.86 | 3.81E-02 |
| 170591 | S100Z [S100 calcium binding protein Z] | -1.86 | 3.19E-02 |
| 120425 | AMICA1 [adhesion molecule, interacts with CXADR antigen 1] | -1.86 | 1.73E-02 |
| 645432 | ARRDC5 [arrestin domain containing 5] | -1.86 | 3.83E-02 |
| 4329 | ALDH6A1 [aldehyde dehydrogenase 6 family, member A1] | -1.87 | 3.38E-02 |
| 79901 | CYBRD1 [cytochrome b reductase 1] | -1.87 | 1.88E-02 |
| 154791 | C7orf55 [chromosome 7 open reading frame 55] | -1.88 | 4.02E-02 |
| 340527 | NHSL2 [NHS-like 2] | -1.89 | 1.96E-02 |
| 912 | CD1D [CD1d molecule] | -1.89 | 2.04E-02 |
| 51449 | PCYOX1 [prenylcysteine oxidase 1] | -1.90 | 1.92E-02 |
| 10497 | UNC13B [unc-13 homolog B (C. elegans)] | -1.90 | 2.28E-02 |
| 6847 | SYCP1 [synaptonemal complex protein 1] | -1.90 | 4.97E-02 |
| 7504 | XK [X-linked Kx blood group (McLeod syndrome)] | -1.91 | 4.12E-02 |
| 1543 | CYP1A1 [cytochrome P450, family 1, subfamily A, polypeptide 1] | -1.91 | 1.43E-02 |
| 154313 | C6orf165 [chromosome 6 open reading frame 165] | -1.91 | 1.69E-02 |
| 2273 | FHL1 [four and a half LIM domains 1] | -1.91 | 2.41E-02 |
| 2535 | FZD2 [frizzled homolog 2 (Drosophila)] | -1.92 | 3.08E-02 |
| 55172 | C14orf104 [chromosome 14 open reading frame 104] | -1.93 | 2.82E-02 |
| 90853 | SPOCD1 [SPOC domain containing 1] | -1.94 | 3.11E-02 |
| 29923 | C7orf68 [chromosome 7 open reading frame 68] | -1.94 | 1.20E-02 |
| 11019 | LIAS [lipoic acid synthetase] | -1.94 | 4.22E-02 |
| 284161 | GDPD1 [glycerophosphodiester phosphodiesterase domain containing 1] | -1.95 | 4.69E-02 |
| 28 | ABO [ABO blood group alpha 1-3-N-acetylgalactosaminyltransferase] | -1.95 | 3.11E-02 |
| 1133 | CHRM5 [cholinergic receptor, muscarinic 5] | -1.98 | 3.53E-02 |
| 1183 | CLCN4 [chloride channel 4] | -1.98 | 2.13E-02 |
| 368 | ABCC6 [ATP-binding cassette, sub-family C (CFTR/MRP), member 6] | -2.00 | 2.15E-02 |
| 117286 | CIB3 [calcium and integrin binding family member 3] | -2.00 | 3.73E-02 |
| 729967 | MORN2 [MORN repeat containing 2] | -2.01 | 3.74E-02 |
| 3752 | KCND3 [potassium voltage-gated channel, Shal-related subfamily, memb 3] | -2.02 | 4.60E-02 |
| 51284 | TLR7 [toll-like receptor 7] | -2.02 | 1.18E-02 |
| 85415 | RHPN2 [rhophilin, Rho GTPase binding protein 2] | -2.03 | 1.83E-02 |
| 10335 | MRVI1 [murine retrovirus integration site 1 homolog] | -2.04 | 2.57E-02 |
| 2147 | F2 [coagulation factor II (thrombin)] | -2.04 | 4.00E-02 |
| 79657 | RPAP3 [RNA polymerase II associated protein 3] | -2.05 | 1.13E-02 |
| 121273 | C12orf54 [chromosome 12 open reading frame 54] | -2.05 | 1.53E-02 |
| 5635 | PRPSAP1 [phosphoribosyl pyrophosphate synthetase-associated protein 1] | -2.05 | 7.10E-03 |
| 27075 | TSPAN13 [tetraspanin 13] | -2.05 | 1.69E-02 |
| 254528 | C16orf73 [chromosome 16 open reading frame 73] | -2.07 | 2.99E-02 |
| 152065 | C3orf22 [chromosome 3 open reading frame 22] | -2.07 | 2.99E-02 |
| 84722 | PSRC1 [proline/serine-rich coiled-coil 1] | -2.07 | 4.31E-02 |
| 2013 | EMP2 [epithelial membrane protein 2] | -2.07 | 3.41E-02 |
| 64073 | C19orf33 [chromosome 19 open reading frame 33] | -2.07 | 4.49E-02 |
| 56034 | PDGFC [platelet derived growth factor C] | -2.08 | 6.72E-03 |
| 400793 | C1orf226 [chromosome 1 open reading frame 226] | -2.09 | 3.62E-02 |
| 9722 | NOS1AP [nitric oxide synthase 1 (neuronal) adaptor protein] | -2.09 | 3.62E-02 |
| 339834 | CCDC36 [coiled-coil domain containing 36] | -2.12 | 2.85E-02 |
| 53630 | BCMO1 [beta-carotene 15,15'-monooxygenase 1] | -2.13 | 4.68E-02 |
| 8739 | HRK [harakiri, BCL2 interacting protein (contains only BH3 domain)] | -2.14 | 3.60E-02 |
| 6445 | SGCG [sarcoglycan, gamma (35kDa dystrophin-associated glycoprotein)] | -2.14 | 2.07E-02 |
| 432 | ASGR1 [asialoglycoprotein receptor 1] | -2.15 | 4.76E-03 |
| 55260 | TMEM143 [transmembrane protein 143] | -2.16 | 1.06E-02 |
| 130888 | FBXO36 [F-box protein 36] | -2.17 | 2.07E-02 |
| 8875 | VNN2 [vanin 2] | -2.18 | 3.25E-03 |
| 7433 | VIPR1 [vasoactive intestinal peptide receptor 1] | -2.19 | 5.31E-03 |
| 83992 | CTTNBP2 [cortactin binding protein 2] | -2.20 | 8.09E-03 |
| 5321 | PLA2G4A [phospholipase A2, group IVA (cytosolic, calcium-dependent)] | -2.20 | 4.77E-03 |
| 171392 | ZNF675 [zinc finger protein 675] | -2.20 | 1.21E-02 |
| 148213 | ZNF681 [zinc finger protein 681] | -2.21 | 4.74E-02 |
| 64757 | MOSC1 [MOCO sulphurase C-terminal domain containing 1] | -2.21 | 3.27E-02 |
| 320 | APBA1 [amyloid beta (A4) precursor protein-binding, family A, member 1] | -2.22 | 3.42E-02 |
| 116369 | SLC26A8 [solute carrier family 26, member 8] | -2.22 | 1.85E-02 |
| 348093 | RBPMS2 [RNA binding protein with multiple splicing 2] | -2.23 | 1.30E-02 |
| 144402 | CPNE8 [copine VIII] | -2.23 | 5.68E-03 |
| 10205 | MPZL2 [myelin protein zero-like 2] | -2.24 | 1.25E-02 |
| 2628 | GATM [glycine amidinotransferase (L-arginine:glycine amidinotransf.)] | -2.25 | 1.05E-02 |
| 10800 | CYSLTR1 [cysteinyl leukotriene receptor 1] | -2.25 | 5.17E-03 |
| 4332 | MNDA [myeloid cell nuclear differentiation antigen] | -2.26 | 1.90E-03 |
| 163786 | SASS6 [spindle assembly 6 homolog (C. elegans)] | -2.29 | 1.67E-02 |
| 166012 | CHST13 [carbohydrate (chondroitin 4) sulfotransferase 13] | -2.29 | 1.95E-02 |
| 10930 | APOBEC2 [apolipoprotein B mRNA editing enzyme, polypeptide-like 2] | -2.29 | 3.97E-02 |
| 55057 | AIM1L [absent in melanoma 1-like] | -2.30 | 1.95E-02 |
| 1028 | CDKN1C [cyclin-dependent kinase inhibitor 1C (p57, Kip2)] | -2.31 | 1.91E-03 |
| 441864 | TARM1 [T cell-interacting, activating receptor on myeloid cells 1] | -2.31 | 1.20E-02 |
| 390927 | ZNF793 [zinc finger protein 793] | -2.32 | 2.01E-02 |
| 27065 | D4S234E [DNA segment on chromosome 4 (unique) 234 expressed seq. | -2.34 | 1.10E-02 |
| 5266 | PI3 [peptidase inhibitor 3, skin-derived] | -2.35 | 1.95E-02 |
| 90627 | STARD13 [StAR-related lipid transfer (START) domain containing 13] | -2.35 | 3.11E-02 |
| 147949 | ZNF583 [zinc finger protein 583] | -2.35 | 1.10E-02 |
| 57664 | PLEKHA4 [pleckstrin homology domain containing, family A member 4] | -2.37 | 1.22E-02 |
| 9156 | EXO1 [exonuclease 1] | -2.38 | 4.59E-02 |
| 65055 | REEP1 [receptor accessory protein 1] | -2.38 | 2.62E-02 |
| 51171 | HSD17B14 [hydroxysteroid (17-beta) dehydrogenase 14] | -2.40 | 7.02E-03 |
| 122945 | C14orf148 [chromosome 14 open reading frame 148] | -2.41 | 2.22E-02 |
| 91646 | TDRD12 [tudor domain containing 12] | -2.42 | 1.82E-02 |
| 2567 | GABRG3 [gamma-aminobutyric acid (GABA) A receptor, gamma 3] | -2.42 | 2.22E-02 |
| 116443 | GRIN3A [glutamate receptor, ionotropic, N-methyl-D-aspartate 3A] | -2.45 | 4.74E-02 |
| 23007 | PLCH1 [phospholipase C, eta 1] | -2.47 | 4.74E-02 |
| 6640 | SNTA1 [syntrophin, alpha 1; dystrophin-associated protein A1] | -2.48 | 3.00E-03 |
| 6247 | RS1 [retinoschisin 1] | -2.48 | 4.81E-02 |
| 2857 | GPR34 [G protein-coupled receptor 34] | -2.48 | 2.74E-03 |
| 116449 | CLNK [cytokine-dependent hematopoietic cell linker] | -2.49 | 4.99E-02 |
| 221264 | AKD1 [adenylate kinase domain containing 1] | -2.49 | 2.39E-03 |
| 389421 | LIN28B [lin-28 homolog B (C. elegans)] | -2.50 | 3.85E-02 |
| 25802 | LMOD1 [leiomodin 1 (smooth muscle)] | -2.50 | 2.05E-02 |
| 283579 | C14orf178 [chromosome 14 open reading frame 178] | -2.51 | 4.81E-02 |
| 2150 | F2RL1 [coagulation factor II (thrombin) receptor-like 1] | -2.51 | 8.59E-03 |
| 79000 | C1orf135 [chromosome 1 open reading frame 135] | -2.55 | 1.59E-02 |
| 7516 | XRCC2 [X-ray repair complementing defective repair in CHO cells 2] | -2.59 | 1.34E-02 |
| 89958 | C9orf140 [chromosome 9 open reading frame 140] | -2.59 | 1.34E-02 |
| 83447 | SLC25A31 [solute carrier family 25 (mitochondrial), member 31] | -2.60 | 3.68E-02 |
| 60675 | PROK2 [prokineticin 2] | -2.62 | 4.19E-03 |
| 10052 | GJC1 [gap junction protein, gamma 1, 45kDa] | -2.63 | 3.23E-02 |
| 219736 | STOX1 [storkhead box 1] | -2.63 | 3.19E-03 |
| 3172 | HNF4A [hepatocyte nuclear factor 4, alpha] | -2.64 | 3.12E-02 |
| 84515 | MCM8 [minichromosome maintenance complex component 8] | -2.67 | 9.28E-04 |
| 100128572 | LOC100128572 [hypothetical LOC100128572] | -2.69 | 2.08E-02 |
| 23025 | UNC13A [unc-13 homolog A (C. elegans)] | -2.70 | 8.06E-03 |
| 130827 | TMEM182 [transmembrane protein 182] | -2.72 | 4.38E-02 |
| 78990 | OTUB2 [OTU domain, ubiquitin aldehyde binding 2] | -2.72 | 2.76E-02 |
| 153090 | DAB2IP [DAB2 interacting protein] | -2.72 | 1.41E-03 |
| 60385 | TSKS [testis-specific serine kinase substrate] | -2.72 | 6.12E-03 |
| 55600 | ITLN1 [intelectin 1 (galactofuranose binding)] | -2.73 | 1.34E-02 |
| 4838 | NODAL [nodal homolog (mouse)] | -2.76 | 2.05E-02 |
| 388815 | C21orf34 [chromosome 21 open reading frame 34] | -2.77 | 1.65E-02 |
| 89874 | SLC25A21 [solute carrier family 25 (mitochondrial), member 21] | -2.77 | 1.34E-02 |
| 79968 | WDR76 [WD repeat domain 76] | -2.78 | 1.57E-03 |
| 26471 | NUPR1 [nuclear protein, transcriptional regulator, 1] | -2.78 | 1.29E-03 |
| 9154 | SLC28A1 [solute carrier family 28, member 1] | -2.80 | 2.81E-02 |
| 7399 | USH2A [Usher syndrome 2A (autosomal recessive, mild)] | -2.80 | 1.66E-02 |
| 51090 | PLLP [plasmolipin] | -2.80 | 1.11E-02 |
| 5957 | RCVRN [recoverin] | -2.81 | 7.74E-03 |
| 970 | CD70 [CD70 molecule] | -2.83 | 1.66E-02 |
| 90381 | C15orf42 [chromosome 15 open reading frame 42] | -2.85 | 6.45E-03 |
| 10595 | ERN2 [endoplasmic reticulum to nucleus signaling 2] | -2.85 | 3.80E-02 |
| 3077 | HFE [hemochromatosis] | -2.86 | 1.05E-03 |
| 50700 | RDH8 [retinol dehydrogenase 8 (all-trans)] | -2.87 | 1.31E-02 |
| 2843 | GPR20 [G protein-coupled receptor 20] | -2.87 | 3.45E-02 |
| 11043 | MID2 [midline 2] | -2.88 | 2.85E-02 |
| 341676 | NEK5 [NIMA (never in mitosis gene a)-related kinase 5] | -2.89 | 7.33E-04 |
| 126668 | TDRD10 [tudor domain containing 10] | -2.91 | 1.66E-02 |
| 22998 | LIMCH1 [LIM and calponin homology domains 1] | -2.93 | 1.66E-02 |
| 10107 | TRIM10 [tripartite motif-containing 10] | -2.98 | 3.98E-02 |
| 781 | CACNA2D1 [calcium channel, voltage-dependent, alpha 2/delta subunit 1] | -3.03 | 1.67E-02 |
| 4868 | NPHS1 [nephrosis 1, congenital, Finnish type (nephrin)] | -3.05 | 2.88E-02 |
| 58510 | PRODH2 [proline dehydrogenase (oxidase) 2] | -3.05 | 2.88E-02 |
| 84072 | HORMAD1 [HORMA domain containing 1] | -3.07 | 1.07E-02 |
| 81029 | WNT5B [wingless-type MMTV integration site family, member 5B] | -3.07 | 1.73E-03 |
| 6997 | TDGF1 [teratocarcinoma-derived growth factor 1] | -3.08 | 2.87E-02 |
| 64927 | TTC23 [tetratricopeptide repeat domain 23] | -3.08 | 1.32E-02 |
| 8838 | WISP3 [WNT1 inducible signaling pathway protein 3] | -3.08 | 2.87E-02 |
| 9365 | KL [klotho] | -3.09 | 2.12E-02 |
| 27197 | GPR82 [G protein-coupled receptor 82] | -3.11 | 3.72E-03 |
| 388551 | CEACAM16 [carcinoembryonic antigen-related cell adhesion molecule 16] | -3.11 | 7.07E-03 |
| 81930 | KIF18A [kinesin family member 18A] | -3.12 | 3.98E-02 |
| 55143 | CDCA8 [cell division cycle associated 8] | -3.14 | 5.02E-03 |
| 8995 | TNFSF18 [tumor necrosis factor (ligand) superfamily, member 18] | -3.17 | 8.01E-03 |
| 1062 | CENPE [centromere protein E, 312kDa] | -3.18 | 1.08E-03 |
| 6123 | RPL3L [ribosomal protein L3-like] | -3.20 | 3.51E-03 |
| 57158 | JPH2 [junctophilin 2] | -3.23 | 8.55E-03 |
| 1364 | CLDN4 [claudin 4] | -3.25 | 2.96E-04 |
| 79820 | CATSPERB [cation channel, sperm-associated, beta] | -3.25 | 1.21E-02 |
| 285588 | EFCAB9 [EF-hand calcium binding domain 9] | -3.26 | 2.87E-02 |
| 161394 | C14orf174 [chromosome 14 open reading frame 174] | -3.27 | 1.21E-02 |
| 387890 | TMEM233 [transmembrane protein 233] | -3.33 | 4.02E-02 |
| 4430 | MYO1B [myosin IB] | -3.33 | 3.27E-03 |
| 387266 | KRTAP5-3 [keratin associated protein 5-3] | -3.36 | 4.02E-02 |
| 387267 | KRTAP5-4 [keratin associated protein 5-4] | -3.36 | 4.02E-02 |
| 64208 | POPDC3 [popeye domain containing 3] | -3.37 | 4.02E-02 |
| 6457 | SH3GL3 [SH3-domain GRB2-like 3] | -3.42 | 4.02E-02 |
| 55507 | GPRC5D [G protein-coupled receptor, family C, group 5, member D] | -3.42 | 2.87E-02 |
| 644150 | WIPF3 [WAS/WASL interacting protein family, member 3] | -3.44 | 9.92E-03 |
| 23024 | PDZRN3 [PDZ domain containing ring finger 3] | -3.44 | 2.61E-03 |
| 7287 | TULP1 [tubby like protein 1] | -3.48 | 4.02E-02 |
| 84141 | FAM176A [family with sequence similarity 176, member A] | -3.48 | 9.92E-03 |
| 56203 | LMOD3 [leiomodin 3 (fetal)] | -3.48 | 1.15E-02 |
| 4355 | MPP2 [membrane protein, palmitoylated 2 (MAGUK p55 subfamily # 2)] | -3.51 | 8.01E-03 |
| 84665 | MYPN [myopalladin] | -3.53 | 6.78E-03 |
| 148979 | GLIS1 [GLIS family zinc finger 1] | -3.54 | 2.81E-02 |
| 79819 | WDR78 [WD repeat domain 78] | -3.56 | 1.74E-03 |
| 54437 | SEMA5B [semaphorin 5B] | -3.57 | 2.81E-02 |
| 56521 | DNAJC12 [DnaJ (Hsp40) homolog, subfamily C, member 12] | -3.60 | 1.16E-03 |
| 11240 | PADI2 [peptidyl arginine deiminase, type II] | -3.61 | 3.76E-06 |
| 7455 | ZAN [zonadhesin] | -3.63 | 6.89E-05 |
| 4166 | CHST6 [carbohydrate (N-acetylglucosamine 6-O) sulfotransferase 6] | -3.65 | 4.22E-03 |
| 79187 | FSD1 [fibronectin type III and SPRY domain containing 1] | -3.65 | 3.00E-04 |
| 8639 | AOC3 [amine oxidase, copper containing 3 (vascular adhesion protein 1)] | -3.69 | 9.53E-03 |
| 2563 | GABRD [gamma-aminobutyric acid (GABA) A receptor, delta] | -3.69 | 2.81E-02 |
| 160428 | ALDH1L2 [aldehyde dehydrogenase 1 family, member L2] | -3.69 | 7.23E-04 |
| 8516 | ITGA8 [integrin, alpha 8] | -3.70 | 8.60E-04 |
| 6017 | RLBP1 [retinaldehyde binding protein 1] | -3.71 | 1.80E-03 |
| 55576 | STAB2 [stabilin 2] | -3.74 | 1.49E-02 |
| 26575 | RGS17 [regulator of G-protein signaling 17] | -3.75 | 5.09E-03 |
| 153478 | PLEKHG4B [pleckstrin homology domain containing, family G # 4B] | -3.75 | 2.59E-03 |
| 4751 | NEK2 [NIMA (never in mitosis gene a)-related kinase 2] | -3.77 | 1.07E-02 |
| 11131 | CAPN11 [calpain 11] | -3.78 | 5.04E-04 |
| 83540 | NUF2 [NUF2, NDC80 kinetochore complex component, homolog] | -3.80 | 6.21E-03 |
| 1136 | CHRNA3 [cholinergic receptor, nicotinic, alpha 3] | -3.81 | 1.97E-02 |
| 146456 | TMED6 [transmembrane emp24 protein transport domain containing 6] | -3.86 | 5.84E-04 |
| 9228 | DLGAP2 [discs, large (Drosophila) homolog-associated protein 2] | -3.86 | 6.21E-03 |
| 730 | C7 [complement component 7] | -3.86 | 1.97E-02 |
| 417 | ART1 [ADP-ribosyltransferase 1] | -3.92 | 3.96E-02 |
| 23624 | CBLC [Cas-Br-M (murine) ecotropic retroviral transforming sequence c] | -3.95 | 8.04E-06 |
| 80032 | ZNF556 [zinc finger protein 556] | -3.95 | 3.55E-03 |
| 1463 | NCAN [neurocan] | -3.99 | 3.96E-02 |
| 91807 | MYLK3 [myosin light chain kinase 3] | -4.02 | 7.69E-03 |
| 2042 | EPHA3 [EPH receptor A3] | -4.03 | 1.38E-02 |
| 260429 | PRSS33 [protease, serine, 33] | -4.05 | 1.38E-02 |
| 56667 | MUC13 [mucin 13, cell surface associated] | -4.05 | 7.69E-03 |
| 57608 | KIAA1462 [KIAA1462] | -4.09 | 3.37E-03 |
| 54943 | DNAJC28 [DnaJ (Hsp40) homolog, subfamily C, member 28] | -4.10 | 1.10E-03 |
| 342132 | ZNF774 [zinc finger protein 774] | -4.15 | 3.67E-04 |
| 146433 | IL34 [interleukin 34] | -4.20 | 2.68E-02 |
| 2515 | ADAM2 [ADAM metallopeptidase domain 2] | -4.20 | 2.68E-02 |
| 85452 | KIAA1751 [KIAA1751] | -4.21 | 3.37E-05 |
| 347273 | MURC [muscle-related coiled-coil protein] | -4.21 | 9.04E-03 |
| 54682 | MANSC1 [MANSC domain containing 1] | -4.21 | 2.68E-02 |
| 81624 | DIAPH3 [diaphanous homolog 3 (Drosophila)] | -4.24 | 1.62E-03 |
| 84966 | IGSF21 [immunoglobin superfamily, member 21] | -4.27 | 2.68E-02 |
| 129880 | BBS5 [Bardet-Biedl syndrome 5] | -4.29 | 8.18E-04 |
| 284348 | LYPD5 [LY6/PLAUR domain containing 5] | -4.31 | 1.62E-03 |
| 5657 | PRTN3 [proteinase 3] | -4.34 | 2.98E-04 |
| 5669 | PSG1 [pregnancy specific beta-1-glycoprotein 1] | -4.39 | 5.09E-03 |
| 5670 | PSG2 [pregnancy specific beta-1-glycoprotein 2] | -4.39 | 5.09E-03 |
| 5672 | PSG4 [pregnancy specific beta-1-glycoprotein 4] | -4.39 | 5.09E-03 |
| 5680 | PSG11 [pregnancy specific beta-1-glycoprotein 11] | -4.39 | 5.09E-03 |
| 221150 | SKA3 [spindle and kinetochore associated complex subunit 3] | -4.44 | 6.46E-04 |
| 125931 | CEACAM20 [carcinoembryonic antigen-related cell adhesion molecule 20] | -4.49 | 1.23E-02 |
| 342931 | RFPL4A [ret finger protein-like 4A] | -4.53 | 1.82E-02 |
| 11061 | LECT1 [leukocyte cell derived chemotaxin 1] | -4.53 | 4.75E-03 |
| 9971 | NR1H4 [nuclear receptor subfamily 1, group H, member 4] | -4.59 | 3.75E-02 |
| 27295 | PDLIM3 [PDZ and LIM domain 3] | -4.62 | 2.88E-03 |
| 169611 | OLFML2A [olfactomedin-like 2A] | -4.64 | 2.88E-03 |
| 8382 | NME5 [nucleoside-diphosphate kinase expressed in non-metastatic cells 5] | -4.64 | 1.71E-04 |
| 159686 | CCDC147 [coiled-coil domain containing 147] | -4.65 | 2.88E-03 |
| 84518 | CNFN [cornifelin] | -4.69 | 3.75E-02 |
| 51704 | GPRC5B [G protein-coupled receptor, family C, group 5, member B] | -4.74 | 4.75E-03 |
| 9099 | USP2 [ubiquitin specific peptidase 2] | -4.79 | 8.33E-03 |
| 65987 | KCTD14 [potassium channel tetramerisation domain containing 14] | -4.81 | 1.33E-04 |
| 100132074 | FOXO6 [forkhead box O6] | -4.83 | 1.23E-02 |
| 142683 | ITLN2 [intelectin 2] | -4.84 | 1.23E-02 |
| 845 | CASQ2 [calsequestrin 2 (cardiac muscle)] | -4.94 | 3.75E-02 |
| 4325 | MMP16 [matrix metallopeptidase 16 (membrane-inserted)] | -4.97 | 3.75E-02 |
| 27293 | SMPDL3B [sphingomyelin phosphodiesterase, acid-like 3B] | -5.01 | 5.52E-04 |
| 3745 | KCNB1 [potassium voltage-gated channel, Shab-related subfamily, member 1] | -5.02 | 3.75E-02 |
| 142827 | C10orf129 [chromosome 10 open reading frame 129] | -5.08 | 2.43E-02 |
| 9152 | SLC6A5 [solute carrier family 6, member 5] | -5.20 | 2.43E-02 |
| 140883 | ZNF280B [zinc finger protein 280B] | -5.21 | 3.21E-05 |
| 147710 | LOC147710 [hypothetical protein LOC147710] | -5.38 | 5.66E-03 |
| 441430 | ANKRD20A2 [ankyrin repeat domain 20 family, member A2] | -5.41 | 5.66E-03 |
| 56999 | ADAMTS9 [ADAM metallopeptidase with thrombospondin type 1 motif, 9] | -5.43 | 2.43E-02 |
| 9609 | RAB36 [RAB36, member RAS oncogene family] | -5.48 | 2.43E-02 |
| 56165 | TDRD1 [tudor domain containing 1] | -5.55 | 5.75E-04 |
| 286753 | TUSC5 [tumor suppressor candidate 5] | -5.66 | 1.57E-02 |
| 81551 | STMN4 [stathmin-like 4] | -5.77 | 1.57E-02 |
| 221395 | GPR116 [G protein-coupled receptor 116] | -5.78 | 1.57E-02 |
| 2195 | FAT1 [FAT tumor suppressor homolog 1 (Drosophila)] | -5.85 | 1.57E-02 |
| 441425 | ANKRD20A3 [ankyrin repeat domain 20 family, member A3] | -5.91 | 2.62E-03 |
| 347527 | ARSH [arylsulfatase family, member H] | -5.93 | 1.57E-02 |
| 332 | BIRC5 [baculoviral IAP repeat-containing 5] | -5.97 | 8.28E-04 |
| 389197 | C4orf50 [chromosome 4 open reading frame 50] | -6.06 | 5.88E-04 |
| 131405 | TRIM71 [tripartite motif-containing 71] | -6.13 | 7.21E-05 |
| 284339 | TMEM145 [transmembrane protein 145] | -6.23 | 5.43E-05 |
| 2134 | EXTL1 [exostoses (multiple)-like 1] | -6.26 | 2.62E-03 |
| 1644 | DDC [dopa decarboxylase (aromatic L-amino acid decarboxylase)] | -6.35 | 1.04E-06 |
| 401036 | ASB18 [ankyrin repeat and SOCS box-containing 18] | -6.39 | 1.79E-03 |
| 79782 | LRRC31 [leucine rich repeat containing 31] | -6.42 | 1.02E-02 |
| 6439 | SFTPB [surfactant protein B] | -6.43 | 3.79E-05 |
| 162333 | MARCH10 [membrane-associated ring finger (C3HC4) 10] | -6.64 | 2.98E-04 |
| 149643 | C1orf227 [chromosome 1 open reading frame 227] | -6.68 | 1.59E-05 |
| 7652 | ZNF99 [zinc finger protein 99] | -6.95 | 5.27E-08 |
| 152404 | IGSF11 [immunoglobulin superfamily, member 11] | -7.39 | 8.69E-06 |
| 2561 | GABRB2 [gamma-aminobutyric acid (GABA) A receptor, beta 2] | -7.52 | 5.80E-04 |
| 7137 | TNNI3 [troponin I type 3 (cardiac)] | -8.02 | 5.70E-05 |
| 654231 | OCM [oncomodulin] | -8.10 | 1.86E-03 |
| 340485 | ACER2 [alkaline ceramidase 2] | -8.12 | 1.93E-04 |
| 100287225 | LOC100287225 [hypothetical LOC100287225] | -8.13 | 1.86E-03 |
| 10281 | DSCR4 [Down syndrome critical region gene 4] | -8.22 | 3.24E-02 |
| 158046 | NXNL2 [nucleoredoxin-like 2] | -8.25 | 3.24E-02 |
| 113457 | TUBA3D [tubulin, alpha 3d] | -8.30 | 1.97E-02 |
| 7278 | TUBA3C [tubulin, alpha 3c] | -8.30 | 1.97E-02 |
| 1358 | CPA2 [carboxypeptidase A2 (pancreatic)] | -8.61 | 1.22E-03 |
| 157313 | CDCA2 [cell division cycle associated 2] | -8.84 | 8.40E-07 |
| 176 | ACAN [aggrecan] | -8.86 | 1.22E-03 |
| 3489 | IGFBP6 [insulin-like growth factor binding protein 6] | -8.99 | 1.97E-02 |
| 414149 | ACBD7 [acyl-CoA binding domain containing 7] | -9.25 | 6.62E-05 |
| 2348 | FOLR1 [folate receptor 1 (adult)] | -9.92 | 1.20E-02 |
| 257629 | ANKS4B [ankyrin repeat and sterile alpha motif domain containing 4B] | -10.00 | 3.58E-04 |
| 6770 | STAR [steroidogenic acute regulatory protein] | -10.10 | 1.62E-06 |
| 146167 | SLC38A8 [solute carrier family 38, member 8] | -10.80 | 7.34E-03 |
| 7401 | CLRN1 [clarin 1] | -10.80 | 7.34E-03 |
| 2520 | GAST [gastrin] | -10.90 | 7.34E-03 |
| 4969 | OGN [osteoglycin] | -11.00 | 1.61E-04 |
| 1129 | CHRM2 [cholinergic receptor, muscarinic 2] | -11.60 | 4.52E-03 |
| 389118 | CDHR4 [cadherin-related family member 4] | -12.10 | 1.17E-06 |
| 129025 | ZNF280A [zinc finger protein 280A] | -13.10 | 2.35E-05 |
| 144453 | BEST3 [bestrophin 3] | -13.40 | 1.74E-03 |
| 84253 | GARNL3 [GTPase activating Rap/RanGAP domain-like 3] | -13.40 | 2.07E-17 |
| 57111 | RAB25 [RAB25, member RAS oncogene family] | -13.40 | 1.74E-03 |
| 401089 | C3orf72 [chromosome 3 open reading frame 72] | -16.60 | 4.35E-04 |
| 347088 | GPR144 [G protein-coupled receptor 144] | -18.00 | 1.78E-04 |
| 83543 | AIF1L [allograft inflammatory factor 1-like] | -20.50 | 7.41E-05 |
| 387332 | TBPL2 [TATA box binding protein like 2] | -22.20 | 2.76E-14 |
| 6954 | TCP11 [t-complex 11 homolog (mouse)] | -23.20 | 1.38E-05 |
| 9890 | LPPR4 [lipid phosphate phosphatase-related protein type 4] | -27.00 | 4.12E-06 |
