# Supplementary material for: Synthetic Cationic Peptide IDR-1018 Modulates Human Macrophage Differentiation
Source: PLoS One. 2013 Jan 7;8(1):e52449. doi: 10.1371/journal.pone.0052449 (PMC3538731; doi:10.1371/journal.pone.0052449)
Supplement: Table S3 — M2 subset of IDR-1018 transcriptional data integrated with IRF-4 binding sites. M2-phenotype associated genes containing IRF-4 binding sites that were differentially expressed in human monocytes stimulated with 20 µg/ml IDR-1018. Genes highlighted/undelined demonstrated IRF4 binding within the annotated gene's structure. (DOCX) [file pone.0052449.s006.docx]

**Table S3. M2 subset of IDR-1018 transcriptional data integrated with IRF-4 binding sites:** M2-phenotype associated genes containing IRF-4 binding sites that were differentially expressed in human monocytes stimulated with 20 μg/ml IDR-1018. Genes highlighted/undelined demonstrated IRF4 binding within the annotated gene’s structure.

| **ENTREZ GENE ID** | **Gene** | **Description** | **Fold change**  **(1018)** | **P value**  **(1018)** |
| --- | --- | --- | --- | --- |
| **11095** | ADAMTS8 | ADAM metallopeptidase with thrombospondin type 1 motif, 8 | 11.10 | 3.58E-04 |
| **10253** | SPRY2 | sprouty homolog 2 (Drosophila) | 8.83 | 8.70E-10 |
| **2119** | ETV5 | ets variant 5 | 4.92 | 1.08E-04 |
| **8190** | MIA | melanoma inhibitory activity | 4.39 | 3.96E-02 |
| **25907** | TMEM158 | transmembrane protein 158 (gene/pseudogene) | 3.95 | 7.66E-07 |
| **10693** | **CCT6B** | chaperonin containing TCP1, subunit 6B (zeta 2) | 3.18 | 9.53E-03 |
| **3624** | **INHBA** | inhibin, beta A | 3.08 | 6.83E-04 |
| **1959** | EGR2 | early growth response 2 | 2.91 | 5.83E-05 |
| **3659** | IRF1 | interferon regulatory factor 1 | 2.86 | 6.89E-05 |
| **5743** | PTGS2 | prostaglandin-endoperoxide synthase 2 | 2.79 | 1.17E-04 |
| **3783** | **KCNN4** | potassium interm/small conduct. calcium-activ channel, s N, m 4 | 2.57 | 3.99E-04 |
| **6004** | RGS16 | regulator of G-protein signaling 16 | 2.56 | 4.99E-02 |
| **22822** | **PHLDA1** | pleckstrin homology-like domain, family A, member 1 | 2.51 | 4.70E-04 |
| **7124** | TNF | tumor necrosis factor | 2.35 | 1.46E-03 |
| **10630** | PDPN | podoplanin | 2.33 | 1.22E-02 |
| **4318** | **MMP9** | matrix metallopeptidase 9 | 2.31 | 1.42E-03 |
| **5292** | PIM1 | pim-1 oncogene | 2.31 | 1.63E-03 |
| **284119** | **PTRF** | polymerase I and transcript release factor | 2.29 | 2.68E-03 |
| **6772** | **STAT1** | signal transducer and activator of transcription 1, 91kDa | 2.26 | 1.91E-03 |
| **3939** | LDHA | lactate dehydrogenase A | 2.21 | 2.44E-03 |
| **8651** | SOCS1 | suppressor of cytokine signaling 1 | 2.17 | 7.25E-03 |
| **5328** | PLAU | plasminogen activator, urokinase | 2.08 | 1.63E-02 |
| **7071** | KLF10 | Kruppel-like factor 10 | 2.05 | 6.45E-03 |
| **7035** | TFPI | tissue factor pathway inhibitor | 2.03 | 3.63E-02 |
| **3640** | **INSL3** | insulin-like 3 (Leydig cell) | 2.00 | 4.64E-02 |
| **9021** | SOCS3 | suppressor of cytokine signaling 3 | 1.90 | 1.42E-02 |
| **5214** | **PFKP** | phosphofructokinase, platelet | 1.85 | 1.87E-02 |
| **27074** | LAMP3 | lysosomal-associated membrane protein 3 | 1.84 | 4.14E-02 |
| **4360** | **MRC1** | mannose receptor, C type 1 | 1.84 | 3.93E-02 |
| **8829** | NRP1 | neuropilin 1 | 1.84 | 2.13E-02 |
| **80176** | SPSB1 | splA/ryanodine receptor domain and SOCS box containing 1 | 1.82 | 3.57E-02 |
| **64386** | **MMP25** | matrix metallopeptidase 25 | 1.81 | 2.42E-02 |
| **10538** | **BATF** | basic leucine zipper transcription factor, ATF-like | 1.79 | 4.23E-02 |
| **9308** | CD83 | CD83 molecule | 1.79 | 2.49E-02 |
| **9672** | SDC3 | syndecan 3 | 1.77 | 4.19E-02 |
| **10938** | **EHD1** | EH-domain containing 1 | 1.74 | 3.29E-02 |
| **1130** | LYST | lysosomal trafficking regulator | 1.74 | 3.25E-02 |
| **8767** | RIPK2 | receptor-interacting serine-threonine kinase 2 | 1.74 | 3.81E-02 |
| **200734** | **SPRED2** | sprouty-related, EVH1 domain containing 2 | 1.74 | 3.89E-02 |
| **8460** | **TPST1** | tyrosylprotein sulfotransferase 1 | 1.74 | 4.18E-02 |
| **7052** | TGM2 | transglutaminase 2 | 1.73 | 3.72E-02 |
| **24145** | PANX1 | pannexin 1 | 1.72 | 4.50E-02 |
| **56978** | PRDM8 | PR domain containing 8 | 1.72 | 4.54E-02 |
| **6890** | **TAP1** | transporter 1, ATP-binding cassette, sub-family B (MDR/TAP) | 1.72 | 3.63E-02 |
| **10221** | TRIB1 | tribbles homolog 1 (Drosophila) | 1.71 | 3.88E-02 |
| **1958** | **EGR1** | early growth response 1 | 1.70 | 4.01E-02 |
| **22809** | **ATF5** | activating transcription factor 5 | 1.68 | 4.45E-02 |
| **5468** | **PPARG** | peroxisome proliferator-activated receptor gamma | 1.68 | 4.77E-02 |
| **6515** | **SLC2A3** | solute carrier family 2 (facilitated glucose transporter), member 3 | 1.67 | 4.84E-02 |
| **8877** | **SPHK1** | sphingosine kinase 1 | 1.67 | 5.00E-02 |
| **11237** | RNF24 | ring finger protein 24 | -1.68 | 4.88E-02 |
| **1955** | MEGF9 | multiple EGF-like-domains 9 | -1.72 | 3.85E-02 |
| **81537** | SGPP1 | sphingosine-1-phosphate phosphatase 1 | -1.75 | 4.26E-02 |
| **3613** | IMPA2 | inositol(myo)-1(or 4)-monophosphatase 2 | -1.77 | 3.95E-02 |
| **55** | ACPP | acid phosphatase, prostate | -1.79 | 4.27E-02 |
| **9936** | **CD302** | CD302 molecule | -1.79 | 2.72E-02 |
| **23171** | **GPD1L** | glycerol-3-phosphate dehydrogenase 1-like | -1.84 | 4.01E-02 |
| **51449** | PCYOX1 | prenylcysteine oxidase 1 | -1.90 | 1.92E-02 |
| **79657** | RPAP3 | RNA polymerase II associated protein 3 | -2.05 | 1.13E-02 |
| **27075** | TSPAN13 | tetraspanin 13 | -2.05 | 1.69E-02 |
| **84722** | PSRC1 | proline/serine-rich coiled-coil 1 | -2.07 | 4.31E-02 |
| **2628** | GATM | glycine amidinotransferase (L-arginine:glycine amidinotransf.) | -2.25 | 1.05E-02 |
| **4332** | **MNDA** | myeloid cell nuclear differentiation antigen | -2.26 | 1.90E-03 |
| **26471** | NUPR1 | nuclear protein, transcriptional regulator, 1 | -2.78 | 1.29E-03 |
| **970** | CD70 | CD70 molecule | -2.83 | 1.66E-02 |
| **3077** | **HFE** | hemochromatosis | -2.86 | 1.05E-03 |
| **6123** | RPL3L | ribosomal protein L3-like | -3.20 | 3.51E-03 |
| **11240** | **PADI2** | peptidyl arginine deiminase, type II | -3.61 | 3.76E-06 |
| **51704** | **GPRC5B** | G protein-coupled receptor, family C, group 5, member B | -4.74 | 4.75E-03 |
| **65987** | KCTD14 | potassium channel tetramerisation domain containing 14 | -4.81 | 1.33E-04 |
| **27293** | **SMPDL3B** | sphingomyelin phosphodiesterase, acid-like 3B | -5.01 | 5.52E-04 |
